# PIWI Proteins Play an Antiviral Role in Lepidopteran Cell Lines

**DOI:** 10.3390/v14071442

**Published:** 2022-06-30

**Authors:** Dulce Santos, Thomas-Wolf Verdonckt, Lina Mingels, Stijn Van den Brande, Bart Geens, Filip Van Nieuwerburgh, Anna Kolliopoulou, Luc Swevers, Niels Wynant, Jozef Vanden Broeck

**Affiliations:** 1 Research Group of Molecular Developmental Physiology and Signal Transduction, Division of Animal Physiology and Neurobiology, Department of Biology, KU Leuven, Naamsestraat 59, 3000 Leuven, Belgium; thomaswolf.verdonckt@kuleuven.be (T.-W.V.); lina.mingels@kuleuven.be (L.M.); stijn.vandenbrande@kuleuven.be (S.V.d.B.); bart.geens@kuleuven.be (B.G.); niels.wynant@kuleuven.be (N.W.); jozef.vandenbroeck@kuleuven.be (J.V.B.); 2 Laboratory of Pharmaceutical Biotechnology, Gent University, Ottergemsesteenweg 460, 9000 Gent, Belgium; filip.vannieuwerburgh@ugent.be; 3 Insect Molecular Genetics and Biotechnology, Institute of Biosciences and Applications, National Center for Scientific Research “Demokritos”, Aghia Paraskevi Attikis, 153 10 Athens, Greece; a.kolliopoulou@bio.demokritos.gr (A.K.); swevers@bio.demokritos.gr (L.S.)

**Keywords:** Argonaute, Ago3, *Bombyx mori* Macula-like Latent Virus (MLV), Cricket Paralysis Virus (CrPV), Flock House Virus (FHV), immunity, insect, piRNA, RNAi, sRNA, siRNA, Siwi, *Spodoptera frugiperda* Rhabdovirus (RV), virus

## Abstract

Insect antiviral immunity primarily relies on RNAi mechanisms. While a key role of small interfering (si)RNAs and AGO proteins has been well established in this regard, the situation for PIWI proteins and PIWI-interacting (pi)RNAs is not as clear. In the present study, we investigate whether PIWI proteins and viral piRNAs are involved in the immunity against single-stranded RNA viruses in lepidopteran cells, where two PIWIs are identified (Siwi and Ago3). Via loss- and gain-of-function studies in *Bombyx mori* BmN4 cells and in *Trichoplusia ni* High Five cells, we demonstrated an antiviral role of Siwi and Ago3. However, small RNA analysis suggests that viral piRNAs can be absent in these lepidopteran cells. Together with the current literature, our results support a functional diversification of PIWI proteins in insects.

## 1. Introduction

RNA interference is a post-transcriptional gene silencing mechanism mediated by small (s)RNA molecules. In short, sRNAs are incorporated into an RNA-induced silencing complex (RISC), which is then directed to an RNA target via Watson–Crick base pairing. Subsequently, the effector protein of RISC, namely an Argonaute protein, acts to inhibit or degrade the specific transcript, resulting in suppressed gene expression [1].

The superfamily of Argonaute proteins can be divided into two main clades, namely the AGO and the PIWI. In insects, the AGO clade is formed by Ago1 and Ago2, while the PIWI clade consists of a species-specific number of proteins. According to the Argonaute with which the sRNAs interact in the RISC, three main sRNA groups can be classified: micro (mi)RNAs, which interact with Ago1; small interfering (si)RNAs, which interact with Ago2; and PIWI-interacting (pi)RNAs. This classification is further supported by the main biological processes in which these sRNAs are involved: miRNAs mainly control the expression of endogenous genes, being potentially implicated in the regulation of any biological process; siRNAs are important antiviral effectors; and piRNAs protect genomic integrity, by controlling the activity of mobile genetic agents such as transposable elements (TEs) [2,3,4].

The canonical siRNA pathway is triggered by viral double-stranded (ds)RNA that occurs during the viral replication cycle. This dsRNA is recognized by the Dicer2 enzyme and cleaved into 20–22 nucleotide (nt)-long RNA duplexes, which are then unwound (siRNAs) and incorporated into the Ago2-containing RISC. Subsequently, this complex targets complementary viral transcripts and performs their degradation. Therefore, the siRNA pathway is an efficient, sequence-specific and broadly acting antiviral mechanism [5].

On the other hand, the piRNA pathway is well-known for its role in the control of TEs in the germline [6]. This mechanism, as it is described in the model organisms *Drosophila melanogaster* and *Bombyx mori*, is initiated when antisense single-stranded (ss)RNAs are transcribed from genomic piRNA clusters, which are highly rich in transposon remnants. These ssRNA precursors are then processed by multiple factors into mature piRNAs (25–31 nt). These antisense-piRNAs bind to a PIWI protein, Aubergine in *D. melanogaster* and Siwi in *B. mori*, with a uridine enrichment in the first position (1U) [7]—this is the primary piRNA biogenesis. Next, these antisense-piRNAs find the target transposon transcript and cleave it into sense-piRNAs. These molecules will then bind to a second PIWI protein, named Ago3, which cleaves antisense piRNA precursors into new piRNAs. Afterwards, these are loaded on to another PIWI protein, and so forth. This process is designated as the ping-pong amplification cycle—or secondary piRNA biogenesis—and allows only piRNAs from active transposons to be amplified. Since PIWI-mediated cleavage occurs between nucleotides 10 and 11, the sense-piRNAs bound to Ago3 possess a bias towards an adenosine in the tenth position (10A). Therefore, a 1U/10A nucleotide bias is a typical signature of piRNAs produced by amplification via the ping-pong cycle [8,9,10].

Remarkably, the number of PIWI proteins is very variable amongst different species. For instance, *B. mori* (Lepidoptera) and *Acyrthosiphon pisum* (Hemiptera) encode two and ten PIWI proteins, respectively [11,12]. Pronounced differences in the number of *piwi* genes can be observed even within the same insect order, with the dipteran species *D. melanogaster*, *Culex pipiens* and *Aedes aegypti* expressing three, seven and eight PIWIs, respectively [3]. Moreover, except for the PIWI protein Ago3, no obvious orthology is present amongst the different PIWI proteins [4].

Interestingly, in mosquitoes, besides germline transposon control, the piRNA pathway also plays a very important role in antiviral immunity [13]. This is in contrast with what is observed in *D. melanogaster*, where an antiviral role of this pathway could not be detected in adult flies [14]. Accordingly, the expression of *A. aegypti* PIWI proteins is not strictly enriched in the gonads, as is the case in *Drosophila*. In addition, although specific proteins are involved in antiviral and anti-transposon control in *Aedes*, a ping-pong amplification cycle seems to occur in both cases [13,15,16].

Considering the huge biodiversity represented by the class Insecta and the observed variability of the piRNA machinery, we investigated a potential antiviral role of the piRNA pathway in Lepidoptera, an insect order that encompasses species with high economic and social value. For this, we made use of two important lepidopteran cell lines, namely *B. mori* BmN4 and *Trichoplusia ni* High Five cells. Specifically, via loss- and gain-of-function approaches, we demonstrate an antiviral role of Siwi and Ago3 in these cells. Nevertheless, we could only identify viral piRNAs in some databases derived from BmN4 cells, and no viral piRNAs were identified in High Five cells. In this scope, the potential involvement of viral piRNAs in lepidopteran antiviral immunity deserves further attention in future research.

## 2. Materials and Methods

### 2.1. Sequence Retrieval, Protein Domain Prediction and Phylogenetic Analysis

By using the PIWI sequences of other insects as a reference, namely *D. melanogaster* and *B. mori*, transcript sequence information for *T. ni siwi* and *ago3* was retrieved from NCBI, accession number SRA057390 [17], with tBLASTn. The deduced amino acid sequences were determined using the ExPASy translate tool, which were then used for the prediction of protein domains using the NCBI Conserved Protein Domain Search. Next, complete protein sequences were aligned using ClustalX [18] and used for the construction of a maximum likelihood phylogenetic tree using IQ-Tree version 1.6.12 [19]. Briefly, the best-fit substitution model was found using ModelFinder [20] (LG + G4) and a maximum likelihood tree and a consensus tree were generated using 10,000 ultrafast bootstraps [21] and a 1000-replicate SH-aLRT test [22]. The phylogenetic tree was rendered using iTol [23] (rooted towards the Ago of *Schizosaccharomyces pombe*, a phylogenetically distant organism).

### 2.2. Cell Culture and Transfections

For the in vitro experiments we used four insect cell stocks. Two different stocks of *T. ni* High Five cells were used, namely one persistently infected with the *B. mori* Macula-like Latent Virus (MLV) and one persistently infected with both the MLV and the Flock House Virus (FHV). One stock of *B. mori* BmN4 cells was used, which is persistently infected with the MLV and the *Spodoptera frugiperda* Rhabdovirus (RV). The *B. mori* Bm5 cells were used as a positive control to the overexpression of Siwi and Ago3. The Bm5 cells were selected for this purpose since we had previously used this experimental setting to successfully overexpress these PIWI proteins in this cell line.

The High Five, BmN4 and Bm5 cell lines were maintained in a complete medium consisting of IPL-41 Insect Medium (Sigma-Aldrich, Bornem, Belgium), supplemented with 10% heat-inactivated fetal bovine serum (Sigma-Aldrich, Bornem, Belgium), 0.25 μg/mL of amphotericin B (Sigma-Aldrich, Bornem, Belgium), 100 U/mL penicillin and 100 μg/mL streptomycin (Gibco, Life Technologies, Merelbeke, Belgium). The cells were subcultured weekly and maintained at 27.5 °C. The cells were transfected using Escort IV (Sigma-Aldrich, Bornem, Belgium), according to the manufacturer’s instructions. An optimized volume of the transfection reagent was used, namely 3.7 μL and 15 μL per well of a 24- and 6-well plate, respectively. In the overexpression experiments, the expression constructs were transfected overnight, at a concentration of 1 μg/mL. In addition, the pBmIE1 helper plasmid encoding the *ie-1* gene for *B. mori* nuclear polyhedrosis virus was used, at a concentration of 0.3 μg/mL [24]. In the knockdown experiments, the dsRNA was transfected into BmN4 cells at a concentration of 5 μg/mL, and into High Five cells at a concentration of 2.5 μg/mL. Upon overnight incubation, the transfection mix was replaced for the complete medium mentioned above.

### 2.3. Expression Constructs

The constructs for overexpression, namely pEA-Flag-BmSiwi and pEA-BmAgo3-MycHis, as well as pEA-pac, which contains the ORF of puromycin resistance gene (negative control), were obtained according to the methodology described by Kolliopoulou and Swevers [25]. In short, to generate the *Bm*-Siwi expression construct, the complete ORF was amplified by PCR (*Platinum Taq HF* DNA polymerase; Invitrogen, Waltham, MA, USA). The forward primer contained a *Bgl*II-site for cloning in-frame with the N-terminal Flag tag of a modified pEA lepidopteran expression vector [26], as well as a Kozak initiation sequence [27]. Similarly, in order to create an expression construct for *Bm*-Ago3, the ORF was cloned in the pEA-MycHis lepidopteran expression vector [26] after amplification by PCR (*Platinum Taq HF* DNA polymerase; Invitrogen, Waltham, MA, USA). Both the forward and reverse primers contained a *Bgl*II cloning site, while the forward primer contained a Kozak initiation sequence [27] and an ATG start codon. The reverse primer was appropriately designed for in-frame cloning with the C-terminal MycHis tag of the pEA-MycHis vector. Both pEA-Flag-BmSiwi and pEA-BmAgo3-MycHis were verified by sequencing. The primer sequences are indicated in Table S1.

### 2.4. Protein Extract Preparation and Western Blotting

Transfected Bm5 and High Five cells were collected and lysed by sonication (Branson) in a lysis buffer consisting of Tris-Cl 50 mM, NaCl 150 mM, EDTA 1 mM, Triton-X-100 1%, sodium dodecyl sulphate (SDS) 0.5% and Protease Inhibitor Cocktail Tablets Complete (Roche, Basel, Switzerland). Next, the total amount of proteins was quantified by means of a bicinchoninic acid assay, after which 18 μg of each sample were separated by SDS-polyacrylamide gel electrophoresis. Then, the proteins were transferred to a Trans-Blot Turbo Mini PVDF membrane using the Trans-Blot Turbo Blotting System (Bio-Rad, Hercules, CA, USA). The blots were washed and blocked with a 5% skimmed powder milk solution for 2 h. Mouse anti-c-Myc (Cell Signalling, Danvers, MA, USA) and rabbit anti-Flag antibodies (Sigma-Aldrich, Bornem, Belgium) were diluted (1:5000) and incubated with the blots overnight at room temperature. Washing was then performed, followed by 2 h of incubation with Polyclonal Goat Anti-Mouse or Anti-Rabbit Immunoglobulins/HRP (Dako), diluted 1:50,000. Finally, the blots were washed, and the detection was performed with the Super Signal West Dura Extended Duration Subtract kit (Thermo Scientific, Waltham, MA, USA). The chemiluminescent bands were visualized using a ChemiDoc^™^ MP Imaging System with Image Lab Software (Bio-Rad, Hercules, CA, USA).

### 2.5. Synthesis of dsRNA

DsRNA constructs for *B. mori*, *T. ni siwi* and *ago3* were synthesized using the MEGAscript RNAi kit (Ambion, Austin, TX, USA). A DNA template flanked by two T7 promoter sequences was synthesized. For this, a PCR reaction was performed with cDNA of BmN4 or High Five cells, gene-specific primers containing a T7 promoter sequence at the 5′ end (Table S1), and REDTaq mix (Sigma-Aldrich, Bornem, Belgium) as a source of DNA Taq polymerase, dNTPs and PCR buffer. The amplification products were subsequently analysed by 1% agarose gel electrophoresis and then visualized under UV-light with the ProXima 2500 (Isogen Life Science, De Meern, The Netherlands). Moreover, the template sequences were validated by first cloning the fragments into the pCR4-TOPO vector by means of the TOPO TA Cloning Kit for Sequencing (Life technologies, Carlsbad, CA, USA) and subsequently sequencing the inserted DNA fragments by Sanger Sequencing (LGC, Berlin, Germany). The PCR product was used directly as template for production of dsRNA. Synthesis of *luciferase* dsRNA was performed using a pLitmus 28i vector (New England Biolabs, Ipswich, MA, USA) containing a 513 bp fragment from the ORF of firefly *luciferase* [28]. This vector was linearized by *Eco*RI or *Hind*III (Fastdigest, Thermo Scientific, Waltham, MA, USA) and subjected to RNA synthesis. The MEGAscript RNAi kit (Ambion, Austin, TX, USA) was used for the synthesis and further purification of the dsRNA, according to manufacturers’ instructions. Both *luciferase* RNA strands were first synthesized independently before being mixed to anneal, while transcripts made from a single template with opposing T7 promoters (for *siwi* and *ago3*) were hybridized during the transcription reaction. The final dsRNA integrity and concentration were assessed by gel electrophoresis using a 1% agarose gel and by means of a Nanodrop spectrophotometer (Thermo Fisher Scientific, Waltham, MA, USA).

### 2.6. Production and Quantification of Cricket Paralysis Virus

The Cricket Paralysis Virus (CrPV) suspension was produced in cultured *D. melanogaster* S2 cells as previously described [29], and purified by ultracentrifugation in a sucrose cushion. The final viral pellet was resuspended in phosphate-buffered saline (PBS). The viral concentration was determined by transmission electron microscopy, negative staining, by CODA-CERVA (predecessor of Sciensano, Ukkel, Belgium).

### 2.7. RNA Extractions

BmN4 or High Five cells were harvested by means of a cell scraper, pelleted and snap frozen. For qRT-PCR, the RNA was extracted with the miRNeasy Mini Kit (Qiagen, Hilden, Germany), as described in the correspondent protocol. A DNase treatment (RNase-free DNase set, Qiagen, Hilden, Germany) was performed to eliminate potential genomic DNA contamination. Quality and concentration of the extracted RNA were assessed using a Nanodrop spectrophotometer (Thermo Fisher Scientific, Waltham, MA, USA). For sRNA sequencing, sRNA extractions were performed with the miRNeasy Mini kit (Qiagen, Hilden, Germany) according to the manufacturers’ protocol for sRNAs. Quality and concentration of the extracted sRNA molecules were assessed with the 2100 Bioanalyzer Small RNA kit (Agilent, Santa Clara, CA, USA).

### 2.8. Quantitative Real Time PCR (qRT-PCR)

The cDNA synthesis was performed using the PrimeScript First Strand cDNA Synthesis kit (TaKaRa, Kusatsu, Japan) following manufacturer’s specifications. For each experiment, an equal amount of total RNA for every sample was used, between 200 and 500 ng. Upon synthesis, the cDNA was diluted 25.5 times with MilliQ water. The qRT-PCR primers are displayed in Table S1. The efficiency of each primer pair was assessed by designing relative standard curves for gene transcripts with a serial dilution (5×) of cDNA. In addition, a dissociation protocol was performed to detect the presence of primer dimers as well as amplification of a single PCR-product. Each PCR reaction was performed in duplicate and contained 5 μL of SYBR Green solution (Invitrogen, Waltham, MA, USA), 0.375 μL of 10 μM forward primer, 0.375 μL of 10 μM reverse primer and 4.25 μL of cDNA. The stability of candidate housekeeping genes was assessed with the geNorm program [30]. Hence, *Tn-elf4a* and *Tn-ef1a* were measured to normalize the MLV (*B. mori* Macula-like Latent Virus) RNA levels in High Five cells upon knockdown of PIWIs; *Tn-act* and *Tn-gapdh* to normalize the FHV (*Flock House Virus*) RNA levels in High Five cells upon knockdown of PIWIs; *Tn-rps18* and *Tn-elF4a* to normalize the CrPV (Cricket Paralysis Virus) RNA levels in High Five cells upon knockdown of PIWIs; *Tn-act* and *Tn-elF4a* to normalize the CrPV RNA levels upon overexpression of PIWIs; and *Bm-tub* and *Bm-rpl49* to normalize the MLV and RV (*Spodoptera frugiperda Rhabdovirus*) RNA levels in BmN4 cells upon knockdown of PIWIs. In every experiment, a no-template control was included. The PCR reaction was performed and analysed in a 96-well plate using the StepOne System (ABI Prism, Applied Biosystems, Waltham, MA, USA). The relative RNA quantity was calculated according to the delta–delta Ct method.

### 2.9. CrPV Infection of High Five Cells

High Five cells were collected by centrifugation (8 min at 1000 *g*), resuspended in the CrPV suspension, or PBS (control), diluted in IPL-41 Insect Medium (Sigma-Aldrich, Bornem, Belgium) and incubated at room temperature for 2 h in a shaker plate. Multiplicity of infection (MOI)25 or MOI10 was used, meaning 25 or 10 virions per cell. Then, following a washing step in IPL-41 Insect Medium (Sigma-Aldrich, Bornem, Belgium), the cells were resuspended in the above-described complete medium, plated in 24-well plates, and maintained at 27.5 °C. At the selected time points, the cells were harvested by means of a scraper and collected for further analysis.

### 2.10. Viability Assays

The assessment of viability was performed using a trypan blue assay. For this, equal volumes of 0.4% trypan blue solution (Sigma-Aldrich, Bornem, Belgium) and cell suspension were mixed and loaded on a counting chamber. The determination of the number of living and dead cells was performed manually at an inverted microscope (Leitz, Wetzlar, Germany) or with the TC10^™^ Automated Cell Counter (Bio-Rad, Hercules, CA, USA).

### 2.11. sRNA Sequencing and Analysis

The sRNA library preparations and next generation sequencing were performed by the NXTGNT sequencing facility (Ghent University). Briefly, sequencing libraries were prepared with the TailorMix microRNA Sample Preparation Kit V2 (SeqMatic), according to manufacturer’s protocol. Next, these were sequenced on an Illumina HiSeq 2000 system, generating single-end reads of 50 nt. Adapters were removed from the raw reads with BBDuk (part of BBMap suite) [31]. FastQC (version 0.11.8) [32] was used to check the quality and length distribution of the reads. Publicly available sRNA libraries derived from BmN4 cells (Table S2) were downloaded from NCBI via the NCBI SRA Toolkit [10,33,34,35,36]. When needed, the adaptors were trimmed using Cutadapt [37] or BBMap [31]. FastQC was used to check the quality and length distribution of the reads [32]. In order to identify viral genomes, the sRNA libraries were analysed based on the methodology reported by Wu and colleagues (2010) [38]. Briefly, in order to assemble the sRNAs into contigs, the Velvet program was used, with a K-mer value of 17 [39]. The obtained contigs were used to perform a BLASTn search against all publicly available viral genome sequences (downloaded from the NCBI BLAST databases repository on the 16 August 2017) using Blast2GO [40]. To directly map the reads to the viral and transposon sequences, Bowtie2 was used with the sensitive preset parameters described in the corresponding manual [41] (accessions NC_015524.1 (MLV), KF947078.1 (RV), NC_003924.1 (CrPV), AB455813.1 (Aquila) and FJ666125.1 (Tn5B)). Since the RV genome is formed by (-)ssRNA, the complementary genome was used. To avoid general contaminants and degradation products, reads shorter than 16 nt and longer than 36 nt were not included. The data for plots and tables depicting the length distribution, the position distribution and the nucleotide frequency of the reads were obtained in RStudio, with the viRome package, and Microsoft Excel [42,43].

## 3. Results

### 3.1. Siwi and Ago3 Knockdowns Result in Increased Viral RNA Levels in BmN4 Cells

**Figure 1:**
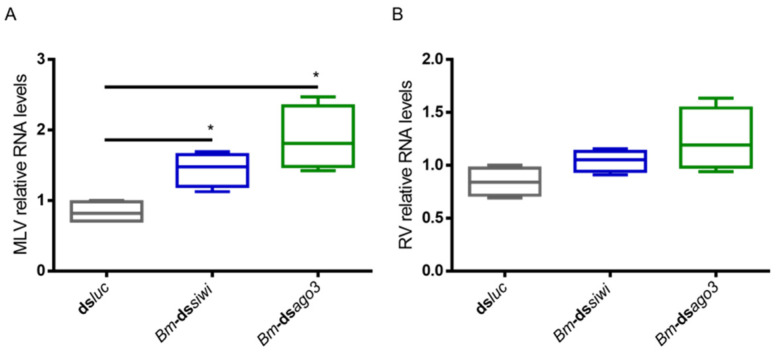
Relative RNA levels of *B. mori* Macula-like Latent Virus (MLV) and *S. frugiperda* Rhabdovirus (RV) in BmN4 cells upon Siwi and Ago3 knockdown. BmN4 cells were transfected with *Bm*-ds*siwi*, *Bm*-ds*ago3* or ds*luc* (negative control). The graphs depict MLV (**A**) and RV (**B**) relative RNA levels, measured by qRT-PCR on day 3 post-transfection. Each box depicts the interquartile range, with the inner line representing the median. The whiskers represent the minimum and maximum values. Statistical analysis (Mann–Whitney test) was performed in GraphPad Prism 6 (* *p* < 0.05; *n* = 4).

We started by investigating a potential antiviral role of the PIWI proteins in BmN4 cells. For this, we induced individual knockdowns of *Bm*-Siwi and *Bm*-Ago3 by delivering transcript-specific dsRNA (*Bm*-ds*siwi* and *Bm*-ds*ago3*) to cells derived from our lab’s stock. A control group was included, to which *luciferase* dsRNA (ds*luc*) was delivered. We then analysed the levels of two viruses known to be persistently infecting our BmN4 cells, namely the *B. mori* Macula-like Latent Virus (MLV) and the *Spodoptera frugiperda* Rhabdovirus (RV). These are, respectively, positive (+) and negative (-)ssRNA viruses [23,24,25,26,27]. The MLV and RV RNA levels were measured by means of qRT-PCR. When dsRNA specific to *siwi* or *ago3* was delivered, increased MLV RNA levels were observed (Figure 1A). Regarding the RV, although a trend was observed in both cases, the differences were not statistically significant (Figure 1B; *p*(ds*luc-Bm*-ds*siwi*) = 0.0571; *p*(ds*luc*-*Bm*-ds*ago3*) = 0.0571). Of note, the dsRNA-induced downregulation of Siwi and Ago3 in these cells was low (Figure S1).

### 3.2. Siwi and Ago3 Knockdowns Result in Increased Viral RNA Levels in High Five cells

**Figure 2:**
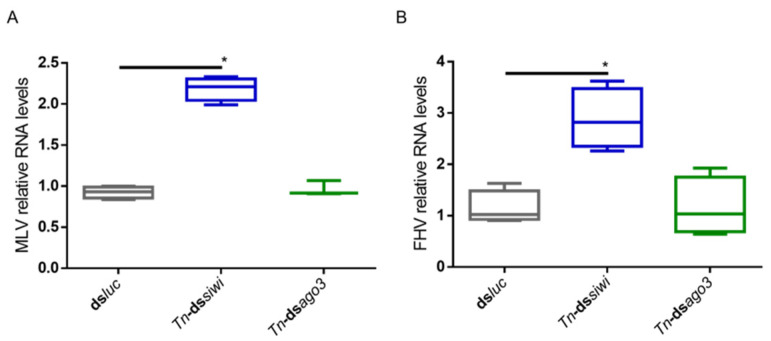
Relative RNA levels of *B. mori* Macula-like Latent Virus (MLV) and Flock House Virus (FHV) in High Five cells upon Siwi and Ago3 knockdown. High Five cells were transfected with *Tn*-ds*siwi*, *Tn*-ds*ago3* or ds*luc* (negative control). The graphs depict MLV (**A**) and FHV (**B**) relative RNA levels, measured by qRT-PCR on day 3 post-transfection. Each box depicts the interquartile range, with the inner line representing the median. The whiskers represent the minimum and maximum values. Statistical analysis (Mann–Whitney test) was performed in GraphPad Prism 6 (* *p* < 0.05; (**A**) *n* ≥ 3; (**B**) *n* = 4).

To investigate if the PIWI proteins play an antiviral role in *T. ni* High Five cells, we induced individual knockdowns of *Tn*-Siwi and *Tn*-Ago3 by delivering transcript-specific dsRNA. The sequences of *Tn-siwi* and *Tn*-*ago3* were first identified from the publicly available transcriptome of High Five cells, and further confirmed via protein domain prediction and phylogenetic analysis (Figure S2) [17]. Ds*luc* was delivered in the control group. We used two High Five cell stocks: one persistently infected with the MLV, where the MLV RNA levels were assessed (Figure 2A); and one persistently infected with both MLV and Flock House Virus (FHV), where the FHV RNA levels were evaluated (Figure 2B). Increased viral RNA levels, measured by qRT-PCR, occurred when Siwi was downregulated. However, no differences were observed when a knockdown of Ago3 was performed (Figure 2).

**Figure 3:**
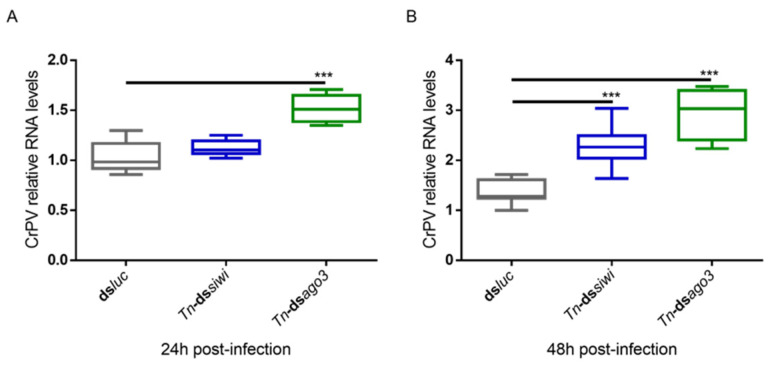
Relative RNA levels of Cricket Paralysis Virus (CrPV) in High Five cells upon Siwi and Ago3 knockdown. High Five cells were transfected with *Tn*-ds*siwi*, *Tn*-ds*ago3* or ds*luc* (negative control) and infected with CrPV (MOI10) on day 3 post-transfection. The graphs depict CrPV relative RNA levels 24 h (**A**) and 48 h (**B**) post-infection, measured by qRT-PCR. Each box depicts the interquartile range, with the inner line representing the median. The whiskers represent the minimum and maximum values. Statistical analysis (Mann–Whitney test) was performed in GraphPad Prism 6 (*** *p* < 0.001; *n* = 8).

To study if PIWI loss-of-function would also lead to higher viral levels for a newly introduced and acute infection, we used the Cricket Paralysis Virus (CrPV). This was performed in High Five cells since these were previously demonstrated to be susceptible to CrPV infection. In contrast to the MLV and FHV persistent infections, the CrPV acute infection is characterized by High Five cell death [44]. In addition, this infection model has previously been successfully used to study the role of Ago2 and Dicer2 in antiviral immunity [44]. Therefore, individual Siwi and Ago3 knockdowns were induced as described above, followed by infection with the CrPV and measurement of the viral RNA levels by qRT-PCR. At 24 h post-infection, elevated viral RNA levels were observed when Ago3 was downregulated (Figure 3A). At 48 h post-infection, elevated CrPV RNA levels were observed when Siwi or Ago3 were downregulated (Figure 3B).

The knockdowns were measured at the transcript level and were efficient for both Siwi and Ago3 (Figure S3).

### 3.3. Overexpression of PIWI Proteins Leads to Reduced CrPV-Induced Mortality

**Figure 4:**
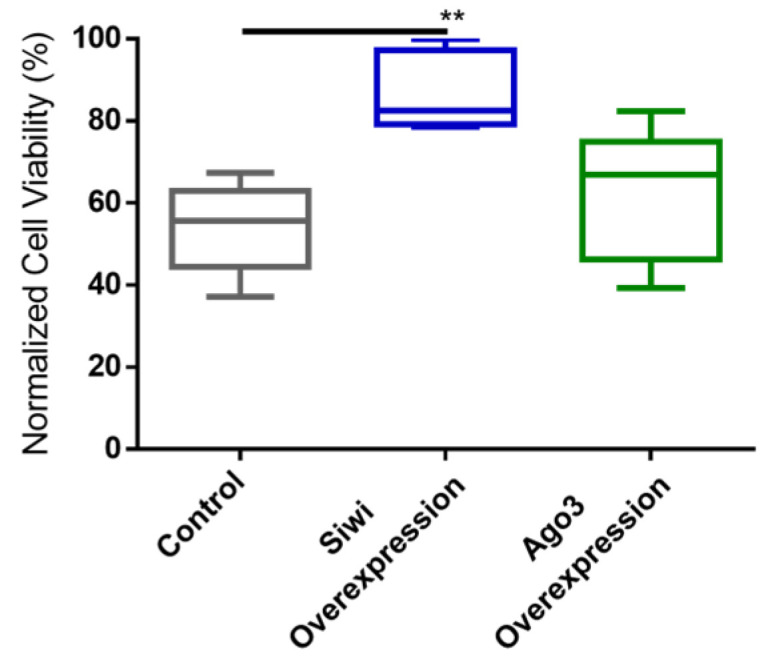
Cricket Paralysis Virus (CrPV)-induced mortality upon overexpression of Siwi and Ago3 in High Five cells. Cells were transfected with overexpression constructs containing the entire ORF of *Bm-siwi* and *Bm-ago3*. The control group was transfected with the pEA-pac control vector containing the ORF of the puromycin resistance gene. The transfected cells were infected with CrPV (MOI25), or treated with PBS, and the viability was assessed after 48 h. The graphs depict the normalized cell viabilities, i.e., viabilities of the CrPV-infected cells normalized to the viabilities of the PBS-treated cells. Each box depicts the interquartile range, with the inner line representing the median. The whiskers represent the minimum and maximum values. Statistical analysis (Mann–Whitney test) was performed in GraphPad Prism 6 (** *p* < 0.005; *n* ≥ 5).

**Figure 5:**
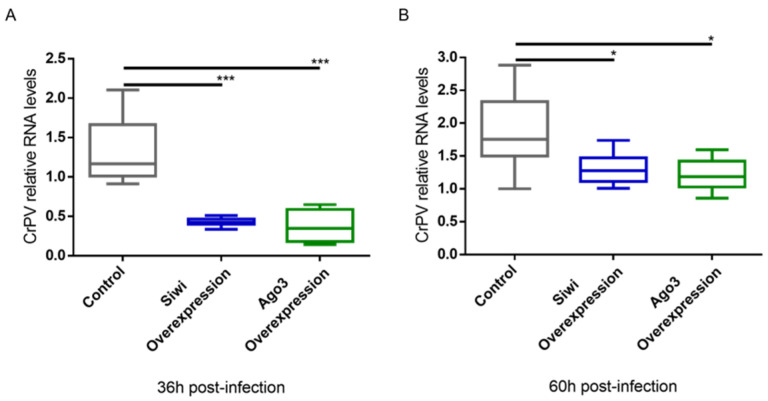
Relative RNA levels of Cricket Paralysis Virus (CrPV) in High Five cells upon overexpression of Siwi and Ago3. Cells were transfected with overexpression constructs containing the entire ORF of *Bm-siwi* and *Bm-ago3*. The control group was transfected with the pEA-pac control vector containing the ORF of the puromycin resistance gene. The transfected cells were infected with CrPV (MOI10) on day 3 post-transfection. The graphs depict CrPV relative RNA levels 36 h (**A**) and 60 h (**B**) post-infection, measured by qRT-PCR. Each box depicts the interquartile range, with the inner line representing the median. The whiskers represent the minimum and maximum values. Statistical analysis (Mann–Whitney test) was performed in GraphPad Prism 6 (*** *p* < 0.001; * *p* < 0.05; *n* = 8).

We further studied the antiviral role of lepidopteran PIWI proteins via a gain-of-function approach. We used the overexpression constructs for *B. mori* (*Bm*)Siwi and *Bm*Ago3 that were already available in our lab [45]. As a negative control, cells were transfected with pEA-pac, which contains the puromycin resistance gene ORF. Every group was subsequently infected with the CrPV, or treated with PBS for subsequent normalization of the cell viability. The overexpression of these proteins was confirmed by a Western blot analysis (Figure S4). In addition, we verified that overexpression of the PIWI proteins did not affect cell viability on their own (Figure S5). Overexpression of Siwi resulted in reduced CrPV-induced mortality (Figure 4). In the case of Ago3, no difference was observed (Figure 4). We continued by assessing if the observed reduced mortality was in line with reduced CrPV levels. In fact, accentuated lower CrPV RNA levels, measured by qRT-PCR, were observed when either Siwi or Ago3 were overexpressed, both at 36 h and 60 h post-infection (Figure 5).

### 3.4. Viral piRNAs Are Only Detected in Some BmN4 Cells-Derived sRNA Databases

**Figure 6:**
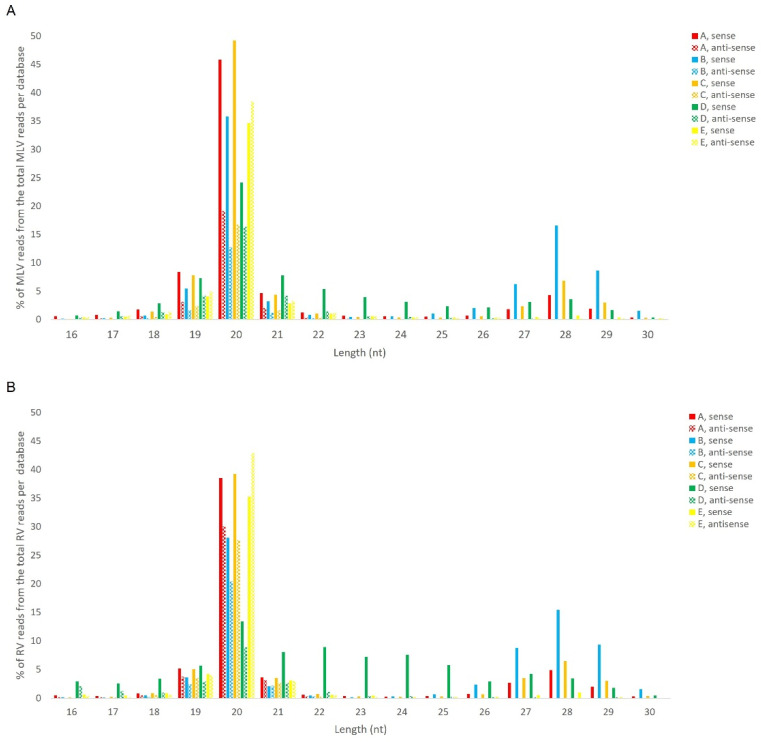
Length distribution of sRNAs, derived from BmN4 cells, mapping to *B. mori* Macula-like Latent Virus (MLV) (**A**) and *S. frugiperda* Rhabdovirus (RV) (**B**). The *Y*-axis represents the percentage of viral reads for each length, in the total of the 16–30 nt viral reads. The *X*-axis represents the length of the reads in nucleotides. Solid bars: sense reads. Dotted bars: anti-sense reads. A–E: represented databases, listed in Table S2.

We analysed the presence of virus-specific piRNAs in *B. mori* BmN4 cells. This cell line was selected since several sRNA libraries are publicly available [10,33,34,35,36]. In addition, the ping-pong piRNA amplification mechanism is known to occur in these cells, with both PIWI proteins being actively expressed [46]. We started by using the publicly available databases indicated in Table S2A–D, consisting of sRNAs from BmN4 cells, obtained by sRNAseq. To identify persistently infecting viruses, sRNA reads were assembled into contigs, which were subsequently used to perform a BLAST search against a database of viruses. This resulted in the identification of two viral genomes, namely the MLV and the RV (by using NCBI BLAST databases downloaded on the 16 August 2017). We then mapped the sRNA reads to these viral genomes and observed that the majority of virus-specific sRNAs are 20 nt-long for both viruses. In addition, a peak at around 28 nt was observed for some databases (Figure 6). Since the total number of reads of the different datasets is very variable due to different sequencing depths, a relative percentage of the viral reads was calculated for the graphic representation. Specifically, considering our aim of comparing the length distributions of the viral sRNAs among databases, the percentage of viral reads for each length, in the total of the 16–30 nt viral reads, is represented (Figure 6). The raw number of viral reads per database is depicted in Table S3. The percentage of viral 28 nt-long RNAs was much lower when compared with the one of 20 nt-long RNAs, and with a clear database-dependent variability. In fact, in some databases, a clear peak at 28 nt is not observed (Figure 6, green). To further investigate this matter, we performed sRNA-sequencing on BmN4 cells derived from our lab’s stock. In this case, for both viruses, a peak of viral sRNAs with the typical length of piRNAs was not detected, while the siRNA peak at 20 nt was evident (Figure 6, yellow).

**Figure 7:**
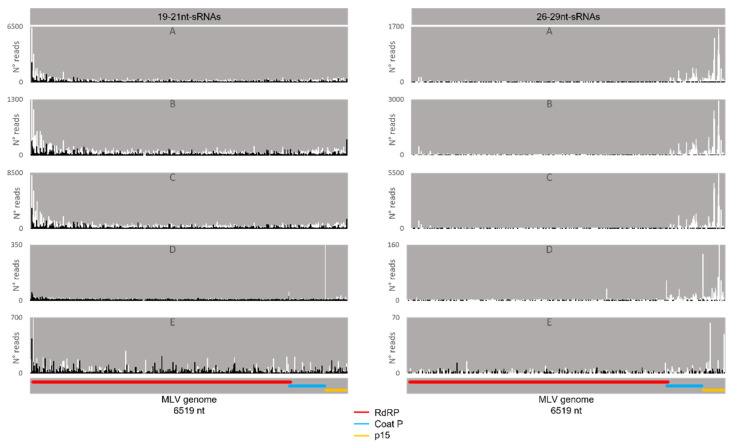
Distribution of BmN4 sRNAs mapping to *B. mori* Macula-like Latent Virus (MLV). Left: distribution of 19–21 nt-long sRNAs. Right: distribution of 26–29 nt-long sRNAs. White bars: sense reads. Black bars: anti-sense reads. A–E: represented databases, listed in Table S2. Red: RdRp ORF; blue: coat P ORF; orange: p15 ORF.

**Figure 8:**
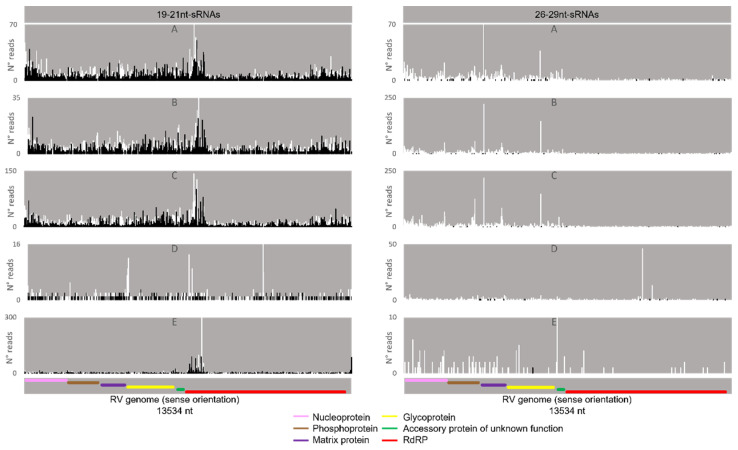
Distribution of BmN4 sRNAs mapping to *S. frugiperda* Rhabdovirus (RV). Left: distribution of sRNAs 19–21 nt-long. Right: distribution of sRNAs 26–29 nt-long. White bars: sense reads. Black bars: anti-sense reads. A–E: represented databases, listed in Table S2. Pink: nucleoprotein ORF; brown: phosphoprotein ORF; purple: matrix protein ORF; yellow: glycoprotein ORF; green: accessory protein of unknown function ORF; red: RdRp ORF.

**Figure 9:**
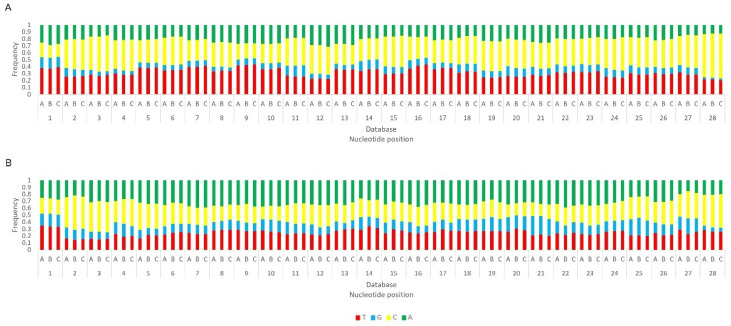
Relative nucleotide frequency of BmN4 28 nt-long sRNAs, mapped to the genome of *B. mori* Macula-like Latent Virus (MLV) (**A**) and *S. frugiperda* Rhabdovirus (RV) (**B**), databases A–C (Table S2). The *Y*-axis represents the relative nucleotide frequency. The *X*-axis represents the position in the read. T: thymine; G: guanine; C: cytosine; A: adenine. Since the sequenced reads correspond to cDNA, thymine corresponds to uracil in the original RNA molecule.

Regarding the orientation of the sRNAs, both sense and antisense 20 nt-long sRNAs were present. However, by contrast, when 28 nt sRNAs were observed, these essentially had a sense orientation (Figure 6, solid and dotted bars). Considering that a bias towards the anti-sense orientation is generally typical of piRNAs, these observations led us to perform a control analysis of our workflow. Specifically, we examined our database by following the same methodologic steps, but, this time, regarding the transposable element Aquila. In this case, the presence of sRNAs with the typical piRNA length and a bias towards the anti-sense orientation were observed as expected, confirming the validity of our workflow (Figure S6). We then analysed where the sRNAs map in the viral sequences. We did this separately for the sRNAs of 19–21 nt (siRNA length range) and of 26–29 nt (piRNA length range). Remarkably, for both viruses, different hotspot regions were observed for the two length ranges (Figure 7 and Figure 8). In addition, we analysed the relative nucleotide frequency of the 28 nt reads mapping to MLV or RV. No obvious nucleotide biases were observed (Figure 9).

**Figure 10:**
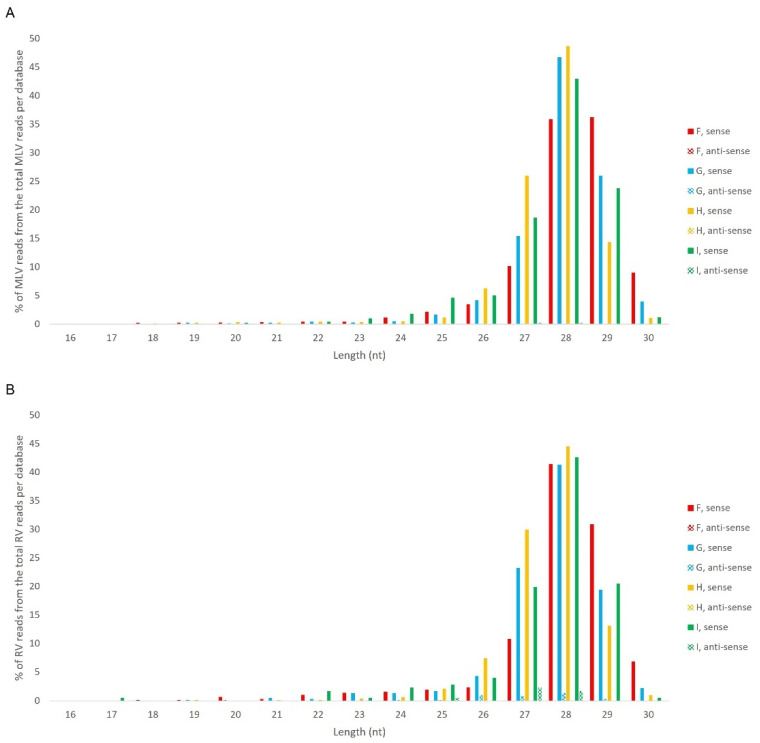
Length distribution of Siwi-bound sRNAs, derived from BmN4 cells, mapping to *B. mori* Macula-like Latent Virus (MLV) (**A**) and *S. frugiperda* Rhabdovirus (RV) (**B**). The *Y*-axis represents the percentage of viral reads for each length, in the total of the 16–30 nt viral reads. The *X*-axis represents the length of the reads in nucleotides. Solid bars: sense reads. Dotted bars: anti-sense reads. F–I: represented databases, listed in Table S2.

**Figure 11:**
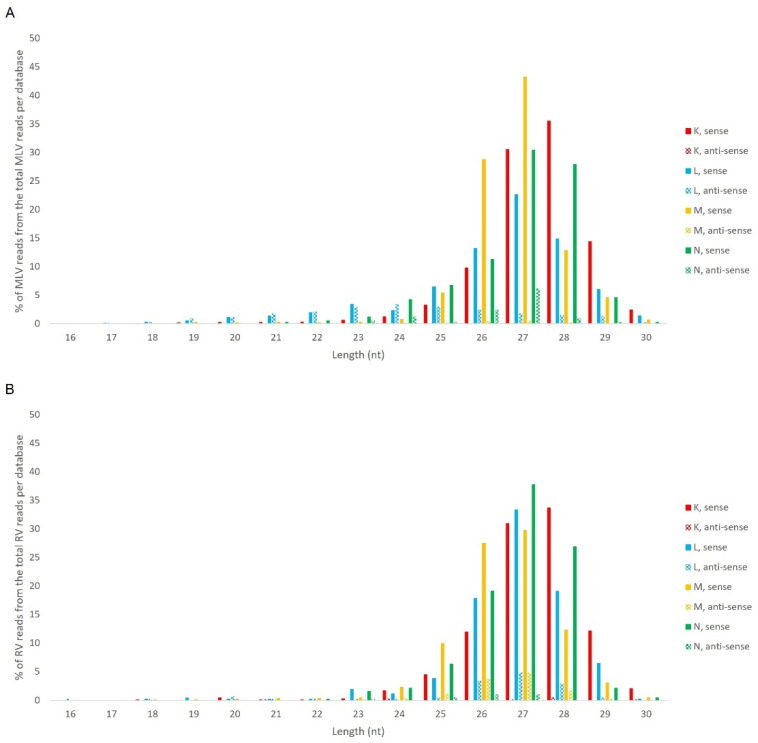
Length distribution of Ago3-bound sRNAs, derived from BmN4 cells, mapping to *B. mori* Macula-like Latent Virus (MLV) (**A**) and *S. frugiperda* Rhabdovirus (RV) (**B**). The *Y*-axis represents the percentage of viral reads for each length, in the total of the 16–30 nt viral reads. The *X*-axis represents the length of the reads in nucleotides. Solid bars: sense reads. Dotted bars: anti-sense reads. F–I: represented databases, listed in Table S2.

Afterwards, we proceeded to investigate whether the viral sRNAs present in BmN4 cells interact with PIWI proteins and can, therefore, be categorically classified as PIWI-interacting (pi)RNAs. To this end, we made use of eight publicly available databases obtained by sRNAseq of Siwi or Ago3 immunoprecipitates (Table S2F–N). This was performed as described above and revealed that, in these BmN4 cells, viral sRNAs can bind to both Siwi and Ago3. Specifically, MLV- and RV-sRNAs were detected in every investigated Siwi and Ago3 immunoprecipitate. Their size corresponded to the typical length of piRNAs, with peaks at 27–28 nt (Figure 10 and Figure 11). Of note, the peak of Siwi-bound viral sRNAs was generally at 28 nt, while the peak of Ago3-bound sRNAs was mainly at 27 nt. Nevertheless, variability among the several databases was observed (Figure 10 and Figure 11). These RNA molecules displayed a clear bias in the strand orientation, with the majority being sense-oriented (Figure 10 and Figure 11). The raw number of viral reads 16–30 nt-long per database is depicted in Table S4.

**Figure 12:**
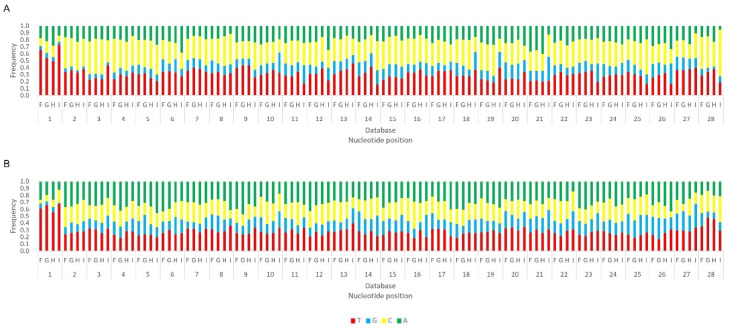
Relative nucleotide frequency of BmN4 Siwi-bound sense 28 nt-long sRNAs, mapped to the genome of *B. mori* Macula-like Latent Virus (MLV) (**A**) and *S. frugiperda* Rhabdovirus (RV) (**B**), databases F–I (Table S2). The *Y*-axis represents the relative nucleotide frequency. The *X*-axis represents the position in the read. T: thymine; G: guanine; C: cytosine; A: adenine. Since the sequenced reads correspond to cDNA, thymine corresponds to uracil in the original RNA molecule.

**Figure 13:**
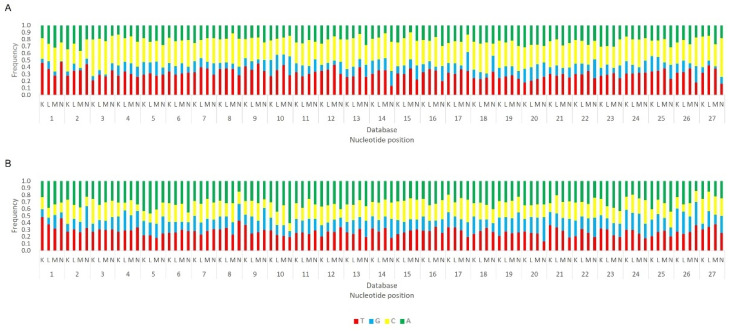
Relative nucleotide frequency of BmN4 Ago3-bound sense 27 nt-long sRNAs, mapped to the genome of *B. mori* Macula-like Latent Virus (MLV) (**A**) and *S. frugiperda* Rhabdovirus (RV) (**B**), databases F–I (Table S2). The *Y*-axis represents the relative nucleotide frequency. The *X*-axis represents the position in the read. T: thymine; G: guanine; C: cytosine; A: adenine. Since the sequenced reads correspond to cDNA, thymine corresponds to uracil in the original RNA molecule.

**Figure 14:**
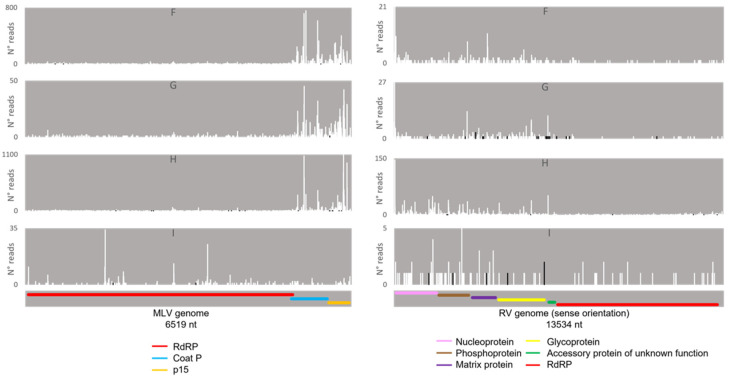
Distribution of 26–29 nt-long sRNAs bound to BmN4 Siwi, mapping to *B. mori* Macula-like Latent Virus (MLV) (**left**) and *S. frugiperda* Rhabdovirus (RV) (**right**). White bars: sense reads. Black bars: anti-sense reads. F–I: represented databases, listed in Table S2. Red: RdRp ORF; blue: coat P ORF; orange: p15 ORF; pink: nucleoprotein ORF; brown: phosphoprotein ORF; purple: matrix protein ORF; yellow: glycoprotein ORF; green: accessory protein of unknown function ORF.

**Figure 15:**
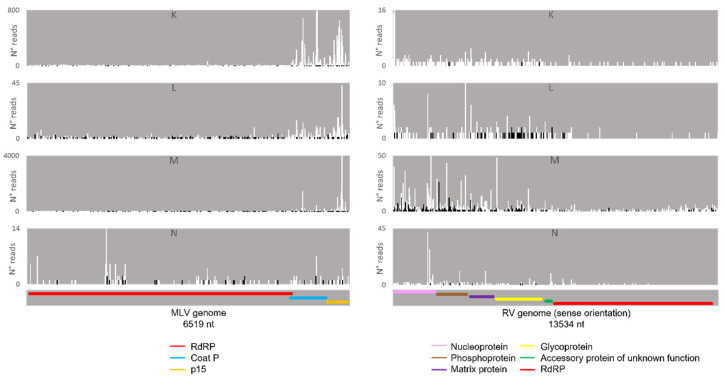
Distribution of 26–29 nt-long sRNAs bound to BmN4 Ago3, mapping to *B. mori* Macula-like Latent Virus (MLV) (**left**) and *S. frugiperda* Rhabdovirus (RV) (**right**). White bars: sense reads. Black bars: anti-sense reads. K–N: represented databases, listed in Table S2. Red: RdRp ORF; blue: coat P ORF; orange: p15 ORF; pink: nucleoprotein ORF; brown: phosphoprotein ORF; purple: matrix protein ORF; yellow: glycoprotein ORF; green: accessory protein of unknown function ORF.

Next, we analysed the nucleotide frequency of the Siwi- and Ago3-bound sRNAs. The sense-stranded Siwi-bound viral piRNAs displayed a bias towards 1U (represented as thymine in the figure, as the data refer to cDNA). This is represented in Figure 12, and was calculated for the sense 28 nt-long RNAs, since these corresponded to the majority of the reads (Figure 10). For the Ago3 viral RNAs, no obvious biases were observed (Figure 13). This was calculated for the sense 27 nt-long RNAs, since these corresponded to the majority of the reads (Figure 11). To control the validity of our workflow, the same analyses were performed for the Aquila transposon in parallel. As expected for transposon-specific piRNAs, a clear bias towards the anti-sense strand was observed in Siwi-bound Aquila sRNAs, while a slight trend to sense sRNAs was observed for Ago3-binding molecules (Figure S7). In addition, clear ping-pong fingerprints were observed, namely noticeable biases towards an uracil in the first position of the Siwi-bound RNAs (represented as thymine in the figure, as the data refer to cDNA); and towards an adenine in the tenth position of the Ago3-bound RNAs (Figure S8). This was calculated for the anti-sense 28 nt Siwi-bound and sense 27 nt Ago3-bound RNAs, respectively, since these corresponded to the majority of the reads (Figure S7). The position to which the viral piRNAs map on the viral genomes was also analysed. One clear hotspot was present in each viral sequence, namely in the terminal third of the MLV genome and in the first half of the RV complementary genome (Figure 14 and Figure 15).

### 3.5. Viral sRNAs with the Typical Length of piRNAs Are Not Detected in High Five Cells

**Figure 16:**
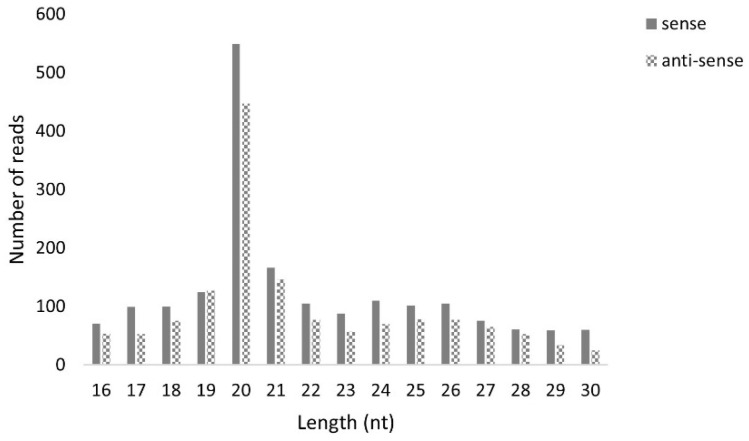
Length distribution of *B. mori* Macula-like Latent Virus (MLV) sRNAs in High Five cells. The *Y*-axis represents the number of reads mapped to MLV. The *X*-axis represents the length of the reads in nucleotides. Solid bars: sense reads. Dotted bars: anti-sense reads.

Next, we studied if MLV sRNAs with the typical length of piRNAs were present in the *T. ni* High Five cell line. For this, sRNAs were purified from our stock of High Five cells, persistently infected with MLV, and sequenced. The obtained reads were then mapped to the MLV genome, revealing one single peak at 20 nt, the siRNA expected length (Figure 16). To verify that this sRNA library included piRNAs, we confirmed the presence of transposon-derived 26–29 nt RNAs, with a strong bias to the anti-sense orientation, for the transposable element Tn5B (Figure S9).

## 4. Discussion

### 4.1. PIWI Proteins Contribute to Antiviral Immunity in BmN4 and High Five Cells

While PIWI proteins and their interacting RNAs are in charge of controlling the activity of TEs, the siRNA pathway is considered the main responsible for insect antiviral immunity [47,48,49,50,51,52]. In this study, we demonstrated that PIWI proteins are involved in the antiviral immunity of two lepidopteran cell lines. We observed that the two lepidopteran PIWIs, namely Siwi and Ago3, affect the outcome of viral infections in the cabbage looper High Five cells and in the silk moth BmN4 cells. In both cell lines, higher viral RNA levels were seen when Siwi or Ago3 were downregulated (Figure 1, Figure 2 and Figure 3). Moreover, reduced CrPV-induced mortality in High Five cells was obtained when Siwi was overexpressed (Figure 4), and lower CrPV levels were seen for the overexpression of both PIWI proteins individually (Figure 5). Of note, no effect was observed on MLV and FHV levels when Ago3 was downregulated in High Five cells (Figure 2), and no reduced CrPV-induced mortality occurred when Ago3 was overexpressed (Figure 4). However, considering our other observations, it is still likely that Ago3 plays an antiviral role. Specifically, in BmN4 cells, higher MLV levels are observed when Ago3 is downregulated (Figure 1); in High Five cells, higher CrPV levels are observed when Ago3 is downregulated (Figure 3), and lower CrPV levels are observed when Ago3 is overexpressed (Figure 5). In this regard, it is possible that Siwi and Ago3 are involved in different steps of virus control, resulting in the observed differences. In addition, it is possible that the employed techniques were not sufficiently sensitive to detect the differences. For instance, it could be that the viral RNA levels were affected at a different time frame, or that the viability assay was of inadequate sensitivity or timing to detect changes. Nonetheless, in line with our observations, Siwi and Ago3 loss-of-function have been previously reported to induce higher MLV RNA levels in BmN4 cells [53]. In addition, the differences we observed for MLV and CrPV equate to those we have previously described for a similar analysis of Dicer2 and Ago2 in High Five cells, main components of the insect antiviral response [44]. Taken together, these observations indicate that both PIWI proteins have an antiviral role in the lepidopteran cells under investigation. Although PIWIs are established to have a key function in mosquito antiviral defence, this is not the case for other insects. In fact, in adult *Drosophila* flies, PIWIs do not seem to be required for this task [14]. These differences are not surprising when noticing the variability in the number of PIWI proteins among different insects. For instance, this family contains four to eight elements in mosquitoes, three in *D. melanogaster*, ten in *A. pisum* (Hemiptera) and two in the described lepidopterans [3,11,12]. This may thus reflect a variation in the range of functions exerted by these proteins.

### 4.2. Viral piRNAs Are Observed in Some BmN4 Stocks but siRNAs Are the Main Class of Viral sRNAs in Lepidopteran Cells

We then investigated the size distribution of the viral sRNAs present in these cell lines. Specifically, 20 nt-long sRNAs, corresponding to the size of siRNAs, form the main class of viral sRNAs in BmN4 and High Five cells (Figure 6 and Figure 16). These observations are in line with what has been previously reported for several viruses in these insect species, supporting that virus-derived siRNAs in *B. mori* and in *T. ni* mainly have a length of 20 nt [54,55,56,57]. Moreover, viral sRNAs with identical length have been demonstrated to bind the Ago2 protein in BmN cells, the parental line of BmN4 cells [44]. Interestingly, in some BmN4 cell-derived databases, a second population of viral sRNAs was observed, characterised by the typical size of piRNAs (Figure 6). In addition, we identified viral sRNAs with the same characteristics in publicly available databases of sRNAs bound to Siwi and Ago3 of BmN4 cells (Figure 10 and Figure 11). Nevertheless, these viral piRNAs were not identified in High Five cells (Figure 16), which is in accordance with what has been previously described [55,56], and were not identified in some databases of BmN4 cells, including the one of our lab (Figure 6). Since these databases were obtained in various laboratories and dates (Table S2), it is likely that these observations reflect differences among distinct stocks of this cell line. In fact, BmN4 cells are reported to readily differentiate into two distinct cell types [55,58], and differences during viral infection of BmN4 cells from three laboratories have been observed [59].

### 4.3. Several Mechanisms Might Contribute to the Generation of the Viral piRNAs Observed in Some BmN4 Stocks

When viral piRNAs are detected, these map to clear hotspots in the viral sequences (Figure 7, Figure 8, Figure 14 and Figure 15). The existence of these hotspots, which are distinct from the ones observed for viral siRNAs (Figure 7 and Figure 8), allow us to hypothesize that viral piRNAs originate via a different mechanism and from a distinct substrate than the viral siRNAs. In addition, viral piRNAs are mainly sense-oriented to the viral ORFs (Figure 6, Figure 10 and Figure 11). Although transposon piRNAs bound to Siwi and Ago3 often present an antisense and sense bias, respectively (Figure S7), this is not a strict rule. In fact, for some transposons, this bias is not present or even inverted [46]. Nevertheless, the fact that the majority of the Siwi- and Ago3- bound viral piRNAs do not show an inverted polarity suggests that the ping-pong amplification mechanism for production of secondary viral piRNAs is not involved here. Interestingly, a tendency for a 1U bias in the sense Siwi-bound viral piRNAs is observed (Figure 12), which is not the case for the viral piRNAs in the whole pool of BmN4 sRNAs (Figure 9). This is in line with previous findings demonstrating a bias of Siwi to bind 1U sRNAs [7]. Regarding the sense viral piRNAs bound to Ago3, these do not present any clear nucleotide bias (Figure 13), making it impossible to predict their origin based on this. Nevertheless, the strong orientation bias of the viral piRNAs binding to Siwi and Ago3 is in accordance with a potential origin from a ssRNA precursor, as is the case for transposon piRNAs [7]. This contrasts with the situation for viral siRNAs (Figure 6), where both sense and anti-sense oriented RNAs were observed since they are mainly generated from long dsRNA precursors [60,61]. Interestingly, biases towards sense viral piRNAs and the presence of hotspot regions have been observed for several arboviruses in *A. aegypti*. These features are not correlated to whether viral genomes are of sense or anti-sense orientation, but they do seem to be conserved between viruses from the same family [13]. Particularly in alphaviruses, these hotspots correlate with subgenomic RNAs [13]. In line with this, the MLV hotspot region is within the subgenomic RNA sequence and includes the ORF of the coat protein (Figure 7, Figure 14 and Figure 15) [62,63]. In addition, a similar hotspot pattern for PIWI-bound MLV sRNAs in BmN4 cells has been previously reported [53]. Regarding the RV hotspot, this includes the ORFs of the abundant structural proteins, i.e., nucleoprotein, phosphoprotein, matrix protein and glycoprotein (Figure 8, Figure 14 and Figure 15) [64]. Therefore, sequences corresponding to viral polymerases are underrepresented in the piRNA population. In this context, it is interesting to hypothesise that the hotspot regions are the result of specific viral transcription and replication strategies and can correlate with high levels of certain (subgenomic) RNAs or of specific viral defective RNAs [65].

MLV piRNAs have been previously reported in BmN4 cells [53]. In addition, viral sRNAs with the typical size of piRNAs have been described in *Tribolium castaneum*, *Diabrotica virgifera*, *Plutella xylostella, A. aegypti* and *B. mori*, as well as in the *D. melanogaster* ovarian somatic sheet (OSS) cells [38,54,66]. Nevertheless, with the exception of *A. aegypti*, the mechanisms of virus-specific piRNA production in insects remain elusive. Recently, viral DNA formation during infection of (non-retroviral) RNA viruses has been observed in dipteran insects, and viral defective RNAs were shown to serve as a template for viral DNA synthesis [67]. These viral DNA molecules were shown to then be used as a template for the production of viral sRNAs [67,68,69,70,71]. Furthermore, viral sequences were detected in every insect genome for which this has been investigated—endogenous viral elements (EVEs)—and different EVEs have been reported in insects from the same species, likely reflecting viral exposure history records in specific populations [72,73,74,75,76,77,78,79]. Of note, RV EVEs have been identified in *S. frugiperda* Sf9 cells [80]. In addition, in several insects, EVEs generate sRNAs, namely si and/or piRNAs [77,81,82], including in *B. mori* [79,81]. In line with this, it is not possible to exclude that the observed differences in viral sRNA length distributions between the different stocks of BmN4 cells (Figure 6) may be due to the acquisition of sRNA-generating MLV and RV EVEs in some of these cell stocks. In parallel, the existence of a PIWI-trigger sequence (PTS) in (ssRNA) piRNA-precursors has recently been reported in *Drosophila* cells. Specifically, a flamenco PTS element is able to trigger piRNA production from the downstream transcript region by recruiting piRNA machinery [34,83]. Therefore, it is interesting to speculate that (some of) these mechanisms might be involved in the generation of specific hotspot patterns of viral piRNAs, as is observed in BmN4 cells.

### 4.4. The Antiviral Action of Lepidopteran PIWI Proteins Might Rely on Several Viral piRNA-Independent Mechanisms

Apart from the situation in mosquitos, the role of viral piRNAs in insects is not established. In this scope, it must be stressed that the functional studies here reported were performed in cells in which viral piRNAs were not detected, namely High Five cells and our stock of BmN4 cells (Figure 1, Figure 2, Figure 3, Figure 4, Figure 5, Figure 6 and Figure 16). As such, these observations make it possible to speculate that, in such scenarios, PIWI proteins might play an antiviral role independent of viral piRNAs. In this context, it is not yet possible to put forward a mechanism by which PIWI proteins are involved in antiviral immunity in these cells. Although this is already well established in mosquitos, the situation is likely to be distinct since (i) mosquitoes have an increased number of PIWIs, varying from four to eight, with distinct specializations, and (ii) viral piRNAs are detected in the soma and germline of these insects, which does not seem to be the case for the investigated lepidopterans [54,55,56,57]. Although no obvious orthology is present amongst PIWIs of different insects, with the exception of Ago3 [4], it is interesting to notice that *A. aegypti* Piwi4 associates with Dicer2 and Ago2 and is a key mediator of the mosquito antiviral mechanism [84,85]. This suggests the potential of some PIWI proteins to associate with members of the siRNA pathway to mount an efficient antiviral response. Remarkably, *A. aegypti* Piwi4 has been demonstrated to preferentially bind EVE-derived piRNAs [84,85]. Therefore, it can be speculated that certain PIWI proteins might have the capacity to recognize sRNAs that originate from distinct precursors. For instance, lepidopteran PIWI proteins could be activated via EVE-derived sRNAs (e.g., pi and/or siRNAs), complementing the role of Dicer2 and Ago2 in antiviral defense via dsRNA-derived sRNAs. Considering the canonical antiviral role of the siRNA-mediated RNAi, as well as the here demonstrated involvement of PIWI proteins in antiviral immunity, it is likely that these mechanisms might functionally interact. Another interesting idea is the existence of an antiviral response of the piRNA pathway, directed by host regulatory piRNAs and independent of viral piRNAs. In fact, several genome-derived piRNAs have recently been found to be differentially expressed in the *B. mori* fat body and midgut upon viral infection [86].

In addition, PIWI proteins and piRNAs are known to direct epigenetic modifications. For instance, they can promote the generation of heterochromatic marks, and some piRNAs are known to be maternally transmitted to the offspring [87]. In the silkworm, a histone methyltransferase is essential for piRNA-mediated sex determination, and PIWI proteins were shown to function as chromatin regulators in BmN4 cells [88,89]. Interestingly, epigenetic mechanisms are reported to be involved in trans-generational immune priming in *Manduca sexta* (Lepidoptera) [90], and it has been recently suggested that genes related to chromatin and DNA binding might be involved in trans-generational antiviral immunity in *Drosophila* [91]. Moreover, a *Caenorhabditis elegans* PIWI-like protein is reported to mediate trans-generational transmission of learned pathogenic avoidance [92]. Therefore, one can speculate that PIWI-mediated epigenetic effects might be involved in the antiviral response of the here studied cell lines.

### 4.5. Soma- and/or Germline-Specific Antiviral PIWI Functions Remain to Be Investigated

When discussing PIWIs and their interacting RNAs, a distinction between the soma and germline needs to be made. Although these proteins are generally highly expressed in the germline and the piRNA ping-pong amplification cycle is active in these cells, it is clear that PIWIs and piRNAs also act in the soma [93]. In *B. mori* in vivo, although the expression of Siwi and Ago3 is highly enriched in the gonads and eggs, the presence of these proteins has been detected in several somatic tissues [11,94]. In BmN4 cells, which are considered germline-derived, both PIWI proteins are known to be actively present [10,33,34,35]. In *T. ni*, PIWIs are expressed in both germline and somatic tissues, but are enriched in the ovaries and testes [55]. Moreover, expression of PIWI proteins was also found to be higher in High Five cells. Although the origin of the cell lines here investigated is not established beyond doubt, BmN4 cells are consensually considered germline-derived [55]. On the other hand, High Five cells have been described to possess hemocyte-like features [95,96,97,98,99]. Interestingly, even though the *B. mori* embryo-derived VF cells were predicted to express *PIWI* genes and piRNAs, no viral piRNAs were detected upon infection with the MLV [55,100]. Therefore, the specific antiviral role of PIWI proteins in the soma and/or germline of lepidopteran insects in vivo remains to be further investigated.

## 5. Conclusions

The current paper assigns a role of the PIWI proteins in antiviral immunity to a group of insects other than mosquitoes. Specifically, we demonstrate an antiviral function for this protein family in lepidopteran cell lines, where only two PIWIs are present. However, the mechanism by which these PIWI proteins exert their antiviral function remains elusive and may not involve viral piRNAs.

## Data Availability

Sequence information of viruses and transposons was retrieved from NCBI, accessions NC_015524.1 (MLV), KF947078.1 (RV), NC_003924.1 (CrPV), AB455813.1 (Aquila) and FJ666125.1 (Tn5B). Transcript sequence information for *T. ni siwi* and *ago3* was retrieved from NCBI, accession SRA057390. The publicly available databases analysed in this study were retrieved from NCBI, SRA numbers SRR5458682, SRR5458683, SRR5458684, DRR079253, SRR1333837, SRR1328015, SRR2034852, SRR1334909, SRR1333838, SRR1328016, SRR2034851 and SRR1334910. The BmN4 and High Five sRNA-Seq datasets obtained during study were deposited on NCBI, SRA numbers SRR17258733 and SRR17258732, respectively.

## Supplementary Materials

The following supporting information can be downloaded at: https://www.mdpi.com/article/10.3390/v14071442/s1, Figures S1–S9; Tables S1–S4. Figure S1: Transcript knockdowns of *Bm*-Siwi and *Bm*-Ago3 in BmN4 cells. BmN4 cells were transfected with *Bm*-ds*siwi*, *Bm*-ds*ago3* or ds*luc* (negative control). The graphs depict *Bm-siwi* (A) and *Bm-ago3* (B) relative transcript levels, measured by qRT-PCR on day 3 post-transfection. Each box depicts the interquartile range, with the inner line representing the median. The whiskers represent the minimum and maximum values (n = 4). Figure S2: Identification of *T. ni* Siwi and Ago3 proteins. (A) Protein domain prediction of *Tn*-Siwi and *Tn*-Ago3. (B) Maximum likelihood phylogenetic tree with the complete amino acid sequence of the known PIWI proteins of *B. mori* (*Bm*-Siwi and *Bm*-Ago3), *D. melanogaster* (*Dm*-Piwi, *Dm*-Aub and *Dm*-Ago3), *Tribolium castaneum* (*Tc*-Aub and *Tc*-Ago3), Helicoverpa armigera (*Ha*-Siwi and *Ha*-Ago3) and *T. ni* (Tn-Siwi and Tn-Ago3); and an Argonaute of *Schizosaccharomyces pombe*, a phylogenetically distant organism (outgroup, *Schp*-Ago). (PAZ: PAZ domain; PIWI: PIWI domain). Figure S3: Transcript knockdowns of *Tn*-Siwi and *Tn*-Ago3 in High Five cells. High Five cells were transfected with *Tn*-ds*siwi*, *Tn*-ds*ago3* or ds*luc* (negative control). The graphs depict *Tn*-siwi (A) and *Tn*-ago3 (B) relative transcript levels, measured by qRT-PCR on day 3 post-transfection. Each box depicts the interquartile range, with the inner line representing the median. The whiskers represent the minimum and maximum values ((A) n = 4; (B) n ≥ 3). Figure S4: Overexpression of *Bm*-Siwi and *Bm*-Ago3 in High Five cells. A Western blot analysis performed with an antibody specific to the FLAG tag (A; *Bm*-Siwi; 101 kDa; blue arrow) or to the Myc tag (B; *Bm*-Ago3; 116 kDa; green arrow) of the expressed proteins confirmed their overexpression. Control cells were transfected with the pEA-pac. Bm5 cells were used as positive control. Lane 1 corresponds to the SeeBlue Plus2 Pre-Stained Protein Standard ladder (Novex). Figure S5: Cell viability upon overexpression of *Bm*-Siwi and *Bm*-Ago3. High Five cells were transfected with a pEA expression vector containing the entire ORF of *Bm*-*siwi* and *Bm*-*ago3*. The control group was transfected with the pEA-pac control vector containing the ORF of puromycin resistance gene. The graphs depict cell viability 3 (A) and 5 (B) days post-transfection. Each box depicts the interquartile range, with the inner line representing the median. The whiskers represent the minimum and maximum values (n = 8). Figure S6: Length distribution of BmN4 sRNAs mapping to Aquila, database E (Table S2). The Y-axis represents the number of reads and the X-axis represents the length of the reads in nucleotides. Solid bars: sense reads. Dotted bars: anti-sense reads. A-E: represented databases, listed in Table S2. Figure S7: Length distribution of BmN4 sRNAs bound to Siwi (A) and Ago3 (B), mapped to Aquila. The Y-axis represents the percentage of viral reads for each length, in the total of the 16–30 nt viral reads. The X-axis represents the length of the reads in nucleotides. Solid bars: sense reads. Dotted bars: anti-sense reads. F–I: represented databases, listed in Table S2. Figure S8: Relative nucleotide frequency of BmN4 sRNAs mapped to Aquila: antisense 28 nt-long sRNAs bound to Siwi (A) and sense 27 nt-long sRNAs bound to Ago3 (B), databases F-N (Table S2). The Y-axis represents the relative nucleotide frequency. The X-axis represents the position in the read. T: thymine; G: guanine; C: cytosine; A: adenine. Since the sequenced reads correspond to cDNA, thymine corresponds to uracil in the original RNA molecule. Figure S9: Length distribution of Tn5B sRNAs in High Five cells. The Y-axis represents the number of reads mapped to the transposable element Tn5B. The X-axis represents the length of the reads in nucleotides. Solid bars: sense reads. Dotted bars: anti-sense reads. Table S1: Primer sequences used for qRT-PCR, for production of dsRNA and for cloning the *B. mori* PIWI proteins. The used T7 promoter sequences are shown in bold and the Kozak initiation sequence is indicated *in italics*. Table S2: Details of the sRNA databases used in this study. Table S3: Length distribution of sRNAs, from databases A-E (Table S2), mapping to MLV or RV. Table S4: Length distribution of sRNAs bound to Siwi or Ago3, from databases F-N (Table S2), mapping to MLV or RV.
