# Supplementary material for: PIWI Proteins Play an Antiviral Role in Lepidopteran Cell Lines
Source: Viruses. 2022 Jun 30;14(7):1442. doi: 10.3390/v14071442 (PMC9321812; doi:10.3390/v14071442)
Supplement: Supplementary file 1 [file viruses-14-01442-s001.zip › viruses-1785899-supplementary.pdf]

Table S1: Primer sequences used for qRT-PCR, for production of dsRNA and for cloning the *B. mori* PIWI proteins. The used T7 promoter sequences are shown in **bold** and the Kozak initiation sequence is indicated *in italics*.

	Forward (5' - 3')	Reverse (5' - 3')
*Bm-siwi*_qRT-PCR	GACAGCACTGGAGTCCGAAA	CGGATCAGGATGCAGACGAT
*Bm-ago3*_ qRT-PCR	GCTTCGGACGCCAACATTAC	TCAGACCGCCTTCGTATTCG
*Bm-αtub*_ qRT-PCR	CTCCCTCCTCCATACCCT	ATCAACTACCAGCCACCC
*Bm-rpl49*_ qRT-PCR	GGTCGTCACTGTGGCTTGG	TCTTCGTCCTGGCCTTCTCA
*Bm-siwi*_T7	**TAATACGACTCACTATAGGGAGA**AATACCCTGCAGGAGACACC	**TAATACGACTCACTATAGGGAGA**GCTACTCATGTTACGAGCGC
*Bm-ago3*_T7	**TAATACGACTCACTATAGGGAGA**CGAAACTGCCAAGAGTCGTC	**TAATACGACTCACTATAGGGAGA**TAATGGCCGCGTATCTGTCG
Bm-siwi	AGTAAGATCT*CAAC*ATGTCAGAACCGAGAGGTAGAG	GTTAGAGGAAATATAAAGTTTCAT
Bm-ago3	ATATAGATCT*CAAC*ATGGCAGACCCAGGCAAAGGC	CCATAGATCTCAAAAAGAACAGCTTATCGACTAAAAC
*Tn-siwi*_ qRT-PCR	CGGCTCCAATGGTTGACCTA	TGTTAGCAGCCAGGAATGCA
*Tn-ago3*_ qRT-PCR	AAGCGTTCCTGTCGTCCAAT	GCTGTCACGTACGAGTCAGT
*Tn-elf4a*_ qRT-PCR	GTGAGCGCGAAGTTATTATGC	AGAAACTTGCTGCACGTCAAT
*Tn-ef1a*_ qRT-PCR	GTCCACAACCACTGGTCACT	TCACGCTCAGCCTTCAGTTT
*Tn-rps18*_ qRT-PCR	GCTCTGTATCGCCGTAACCA	TGCCGTTTCGAACACAAGAC
*Tn-act*_ qRT-PCR	CGCACACGGTGCCCATCTA	CTCGGTGAGGATCTTCATCA
*Tn-gapdh*_qRT-PCR	GCAAGGCTGGTGCTGAATAC	GGCCTTCTCTGTGGTTGTGA
*Tn-siwi*_T7	**TAATACGACTCACTATAGGG**GTTTCCGCATACCCAGACCA	**TAATACGACTCACTATAGGG**TGCAGCAGCCATGTTGAAAC
*Tn-ago3*_T7	**TAATACGACTCACTATAGGG**CGGCGAAATGAAAGTGGTCC	**TAATACGACTCACTATAGGG**TCCGCTAACACTTCAGCAGG
*mlv*_ qRT-PCR	TGAGTTGCGTTCCGAATGGA	ACCAAGACGGGTGCTGATTT
*rv*_ qRT-PCR	CCAACACAGGGACACCATCA	GCAGTCTGTCCTGTGAGCTT
*fhv*_ qRT-PCR	CCAGATCACCCGAACTGAAT	AGGCTGTCAAGCGGATAGAA
*crpv*_ qRT-PCR	ACGAGGAAGCAACTCAAGGA	GAGCCCGCTGAGATGTAAAG

Table S2: Details of the sRNA databases used in this study.

ID in this manuscript	SRA number	Sequencing technology	sRNA Source	Reference
**A**	SRR5458682	ABI SOLiD	BmN4, Thomas Jefferson University	36
**B**	SRR5458683	ABI SOLiD		
**C**	SRR5458684	ABI SOLiD		
**D**	DRR079253	ABI SOLiD	BmN4, University of Tokyo	/
**E**	SRR17258733	Illumina HiSeq	BmN4, KU Leuven (initial stock kindly provided by Prof. Pillai, University of Geneva)	this study
**F**	SRR1333837	Illumina HiSeq	BmN4 Siwi IP, University Grenoble Alpes	10
**G**	SRR1328015	Illumina HiSeq	BmN4 Siwi IP, University Grenoble Alpes	33
**H**	SRR2034852	Illumina HiSeq	BmN4 Siwi IP, University Grenoble Alpes	34
**I**	SRR1334909	Illumina MiSeq	BmN4 Siwi IP, University of Tokyo	35
**K**	SRR1333838	Illumina HiSeq	Ago3 IP, University Grenoble Alpes	10
**L**	SRR1328016	Illumina HiSeq	Ago3 IP, University Grenoble Alpes	33
**M**	SRR2034851	Illumina HiSeq	Ago3 IP, University Grenoble Alpes	34
**N**	SRR1334910	Illumina MiSeq	Ago3 IP, University of Tokyo	35
/	SRR17258732	Illumina HiSeq	High Five, KU Leuven	this study

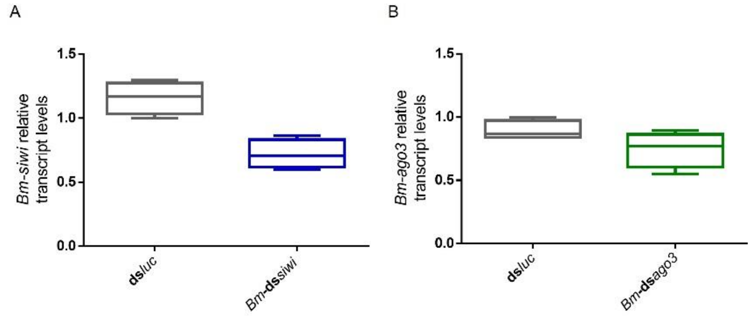

Figure S1: Transcript knockdowns of *Bm-Siwi* and *Bm*-Ago3 in BmN4 cells. BmN4 cells were transfected with *Bm*-ds*siwi*, *Bm*-ds*ago3* or ds*luc* (negative control). The graphs depict *Bm-siwi* (A) and *Bm-ago3* (B) relative transcript levels, measured by qRT-PCR on day 3 post-transfection. Each box depicts the interquartile range, with the inner line representing the median. The whiskers represent the minimum and maximum values (n=4).

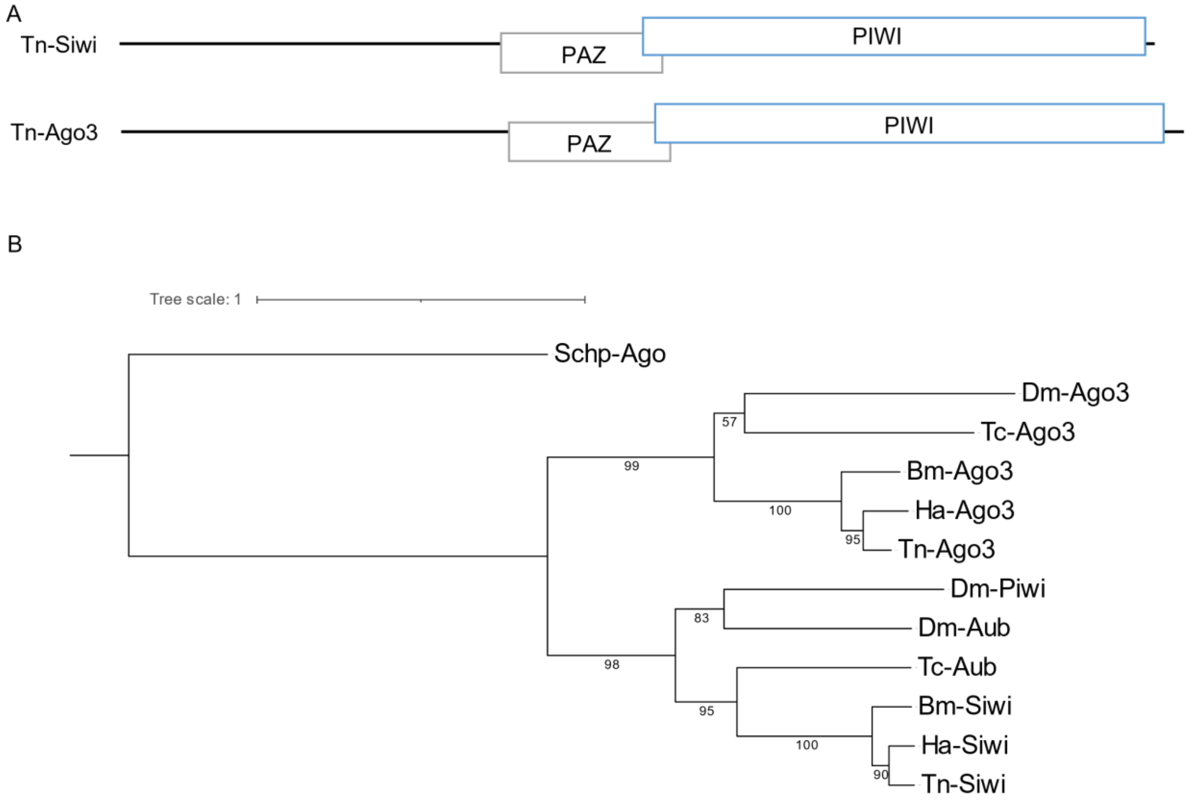

Figure S2: Identification of *T. ni* Siwi and Ago3 proteins. (A) Protein domain prediction of *Tn*-Siwi and *Tn*-Ago3. (B) Maximum likelihood phylogenetic tree with the complete amino acid sequence of the known PIWI proteins of *B. mori* (*Bm*-Siwi and *Bm*-Ago3), *D. melanogaster* (*Dm*-Piwi, *Dm*-Aub and *Dm*-Ago3), *Tribolium castaneum* (*Tc*-Aub and *Tc*-Ago3), *Helicioverpa armigera* (*Ha*-Siwi and *Ha*-Ago3) and *T. ni* (*Tn*-Siwi and *Tn*-Ago3); and an Argonaute of *Schizosaccharomyces pombe*, a phylogenetically distant organism (outgroup, Schp-Ago). (PAZ: PAZ domain; PIWI: PIWI domain).

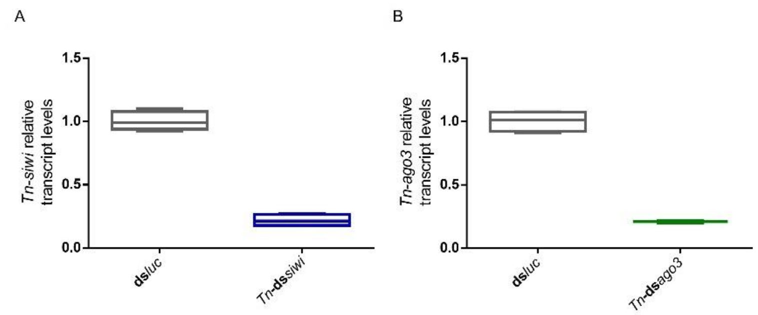

Figure S3: Transcript knockdowns of *Tn*-Siwi and *Tn-Ago3* in High Five cells. High Five cells were transfected with *Tn*-ds*siwi*, *Tn*-ds*ago3* or ds*luc* (negative control). The graphs depict *Tn-siwi* (A) and *Tn-ago3* (B) relative transcript levels, measured by qRT-PCR on day 3 post-transfection. Each box depicts the interquartile range, with the inner line representing the median. The whiskers represent the minimum and maximum values ((A) n=4; (B) n≥3).

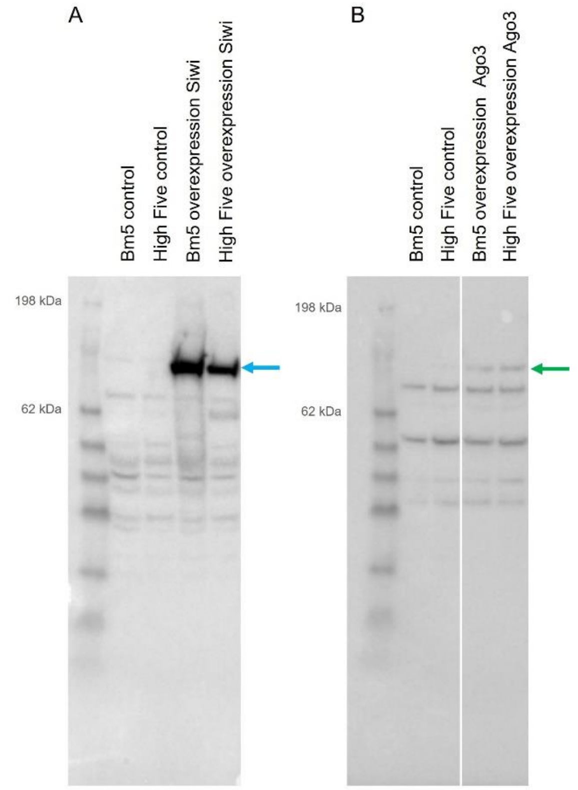

Figure S4: Overexpression of *Bm*-Siwi and *Bm*-Ago3 in High Five cells. A Western blot analysis performed with an antibody specific to the FLAG tag (A; *Bm*-Siwi; 101 kDa; blue arrow) or to the Myc tag (B; *Bm*-Ago3; 116 kDa; green arrow) of the expressed proteins confirmed their overexpression. Control cells were transfected with the pEA-pac. Bm5 cells were used as positive control. Lane 1 corresponds to the SeeBlue Plus2 Pre-Stained Protein Standard ladder (Novex).

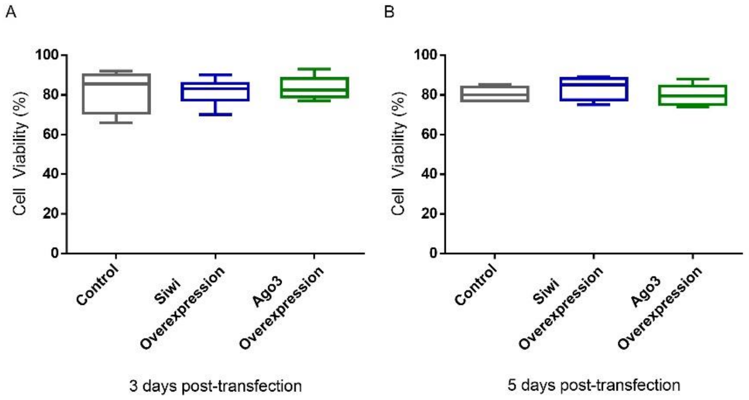

Figure S5: Cell viability upon overexpression of *Bm*-Siwi and *Bm*-Ago3. High Five cells were transfected with a pEA expression vector containing the entire ORF of *Bm-siwi* and *Bm-ago3*. The control group was transfected with the pEA-pac control vector containing the ORF of puromycin resistance gene. The graphs depict cell viability 3 (A) and 5 (B) days post-transfection. Each box depicts the interquartile range, with the inner line representing the median. The whiskers represent the minimum and maximum values (n=8).

Table S3: Length distribution of sRNAs, from databases A-E (Table S2), mapping to MLV or RV.

MLV										
A		B		C		D		E		
sense	anti-sense	sense	anti-sense	sense	anti-sense	sense	anti-sense	sense	anti-sense	Length (nt)
3998	680	376	59	1258	246	296	141	246	297	**16**
5321	1278	598	117	3445	731	574	244	316	423	**17**
11185	3982	1934	603	16829	4792	1116	524	587	875	**18**
54435	20470	15054	4625	94781	29494	2878	1674	2671	3237	**19**
298420	124773	98845	35127	597941	203371	9578	6491	22608	25059	**20**
30311	13307	8756	3310	52976	19515	3076	1649	1850	2173	**21**
8024	2331	2295	674	12790	3814	2128	552	666	779	**22**
4272	784	1242	226	5181	911	1560	226	352	368	**23**
3657	469	1460	153	4190	318	1241	158	227	233	**24**
3249	272	2892	165	3755	187	919	110	205	157	**25**
4524	264	5661	198	6476	151	823	96	170	170	**26**
11758	253	17262	204	28238	233	1219	48	267	120	**27**
27983	195	45712	133	83062	134	1427	40	434	103	**28**
12361	140	23830	72	36174	65	662	24	236	109	**29**
2271	105	4306	90	3553	55	145	11	108	109	**30**

RV										
A		B		C		D		E		
sense	anti-sense	sense	anti-sense	sense	anti-sense	sense	anti-sense	sense	anti-sense	Length (nt)
184	72	33	12	85	12	105	73	55	36	**16**
138	67	17	11	158	73	90	46	40	14	**17**
288	171	83	48	549	349	120	34	74	53	**18**
1745	1271	590	391	3018	2116	203	102	356	331	**19**
13038	10172	4544	3305	23388	16487	477	316	2972	3615	**20**
1222	1077	330	344	2102	1598	288	95	259	248	**21**
208	116	74	58	439	188	319	41	53	41	**22**
130	19	33	3	204	35	258	16	40	14	**23**
99	9	55	12	150	15	271	13	18	7	**24**
125	6	111	6	214	7	204	9	17	1	**25**
263	14	384	11	425	9	104	7	22	0	**26**
906	14	1422	8	2076	17	152	5	47	0	**27**
1647	27	2496	15	3865	9	123	3	87	1	**28**
676	7	1516	7	1797	3	64	5	18	0	**29**
122	2	258	6	226	1	17	1	3	0	**30**

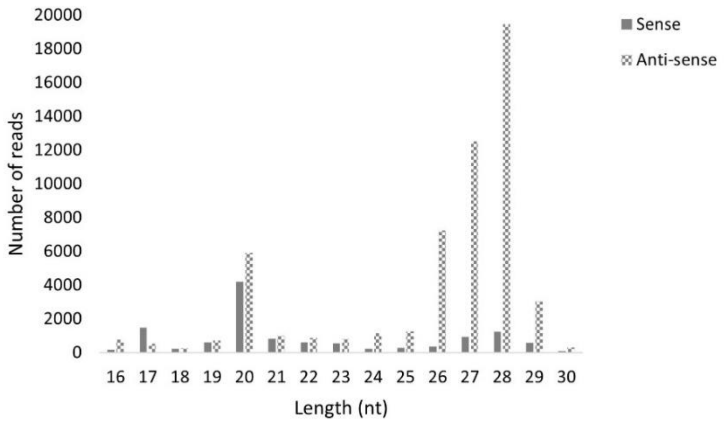

Figure S6: Length distribution of BmN4 sRNAs mapping to Aquila, database E (Table S2). The Y-axis represents the number of reads and the X-axis represents the length of the reads in nucleotides. Solid bars: sense reads. Dotted bars: anti-sense reads. A-E: represented databases, listed in Table S2.

Table S4: Length distribution of sRNAs bound to Siwi or Ago3, from databases F-N (Table S2), mapping to MLV or RV.

MLV-Siwi								
F		G		H		I		Database
Sense	Anti-sense	Sense	Anti-sense	Sense	Anti-sense	Sense	Anti-sense	Length (nt)
2	0	0	0	3	0	0	0	**16**
13	0	1	0	18	0	0	0	**17**
43	0	1	0	27	0	0	0	**18**
56	0	5	0	58	0	0	0	**19**
63	4	3	0	74	0	1	0	**20**
81	0	5	0	67	0	0	0	**21**
99	1	8	0	94	0	2	0	**22**
102	2	6	0	81	0	5	0	**23**
264	0	10	1	115	0	9	0	**24**
498	1	33	0	252	4	23	0	**25**
801	1	83	0	1408	3	25	0	**26**
2378	2	307	0	5837	7	93	1	**27**
8409	0	931	0	10938	5	215	1	**28**
8483	1	518	1	3217	2	119	0	**29**
2118	0	79	0	251	3	6	0	**30**
**RV-Siwi**								
**F**		**G**		**H**		**I**		**Database**
**Sense**	**Anti-sense**	**Sense**	**Anti-sense**	**Sense**	**Anti-sense**	**Sense**	**Anti-sense**	**Length (nt)**
0	0	0	0	0	0	0	0	**16**
0	0	0	0	3	0	1	0	**17**
1	0	0	0	2	0	0	0	**18**
1	0	1	0	7	0	0	0	**19**
4	1	0	0	4	0	0	0	**20**
2	0	3	0	5	0	0	0	**21**
6	0	2	0	6	0	3	0	**22**
8	0	8	0	16	0	1	0	**23**
9	0	8	1	27	0	4	0	**24**
11	0	10	1	87	0	5	1	**25**
13	0	25	6	308	0	7	0	**26**
60	0	135	5	1242	2	35	4	**27**
231	0	240	8	1846	3	75	3	**28**
172	0	113	2	545	2	36	0	**29**
38	0	13	0	40	1	1	0	**30**
**MLV-Ago3**								
**K**		**L**		**M**		**N**		**Database**
**sense**	**anti-sense**	**sense**	**anti-sense**	**sense**	**anti-sense**	**sense**	**anti-sense**	**Length (nt)**
4	0	0	1	4	0	0	0	**16**

7	0	4	4	18	0	0	0	**17**
35	2	8	8	50	2	0	0	**18**
63	1	13	22	118	0	0	0	**19**
87	4	27	26	108	1	0	0	**20**
88	3	32	42	146	5	1	0	**21**
127	4	46	49	96	4	2	0	**22**
208	16	79	66	153	9	4	2	**23**
398	13	55	78	327	57	14	4	**24**
1042	8	148	68	2223	64	22	1	**25**
3069	15	302	57	11660	176	37	8	**26**
9503	22	517	42	17494	211	99	20	**27**
11059	31	339	35	5221	88	91	3	**28**
4513	16	138	29	1862	47	15	1	**29**
770	9	33	5	276	24	1	0	**30**

**RV-Ago3**								
**K**		**L**		**M**		**N**		**Database**
**sense**	**anti-sense**	**sense**	**anti-sense**	**sense**	**anti-sense**	**sense**	**anti-sense**	**Length (nt)**
0	0	0	1	0	0	0	0	**16**
0	0	0	0	0	0	0	0	**17**
1	0	1	1	7	1	0	0	**18**
0	0	2	0	9	0	0	0	**19**
3	0	1	3	12	0	0	0	**20**
1	1	1	1	20	2	0	0	**21**
1	0	1	1	18	2	1	0	**22**
2	0	8	1	27	6	6	1	**23**
10	2	5	1	108	18	8	0	**24**
26	0	16	2	460	52	24	2	**25**
69	0	74	14	1267	174	72	4	**26**
178	1	138	20	1369	220	142	4	**27**
194	3	79	12	568	76	101	0	**28**
70	0	27	2	143	11	8	0	**29**
12	1	1	0	24	3	2	0	**30**

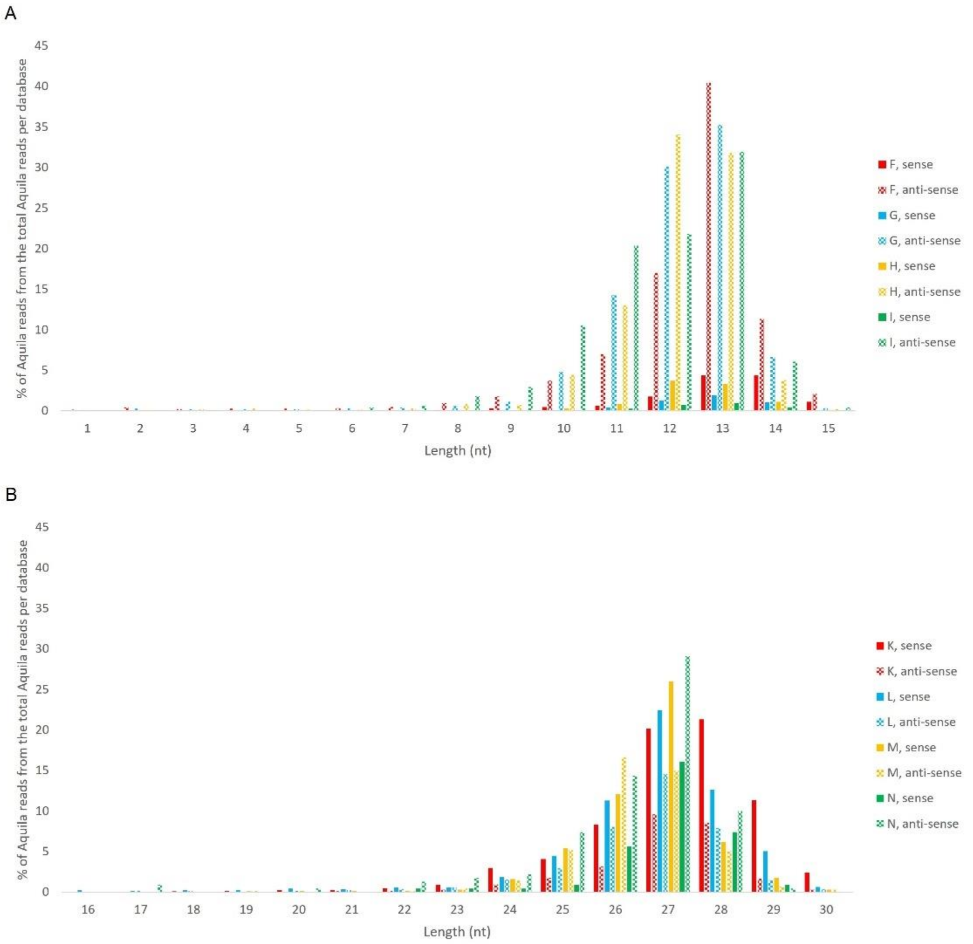

Figure S7: Length distribution of BmN4 sRNAs bound to Siwi (A) and Ago3 (B), mapped to Aquila. The Y-axis represents the percentage of viral reads for each length, in the total of the 16-30nt viral reads. The X-axis represents the length of the reads in nucleotides. Solid bars: sense reads. Dotted bars: anti-sense reads. F-I: represented databases, listed in Table S2.

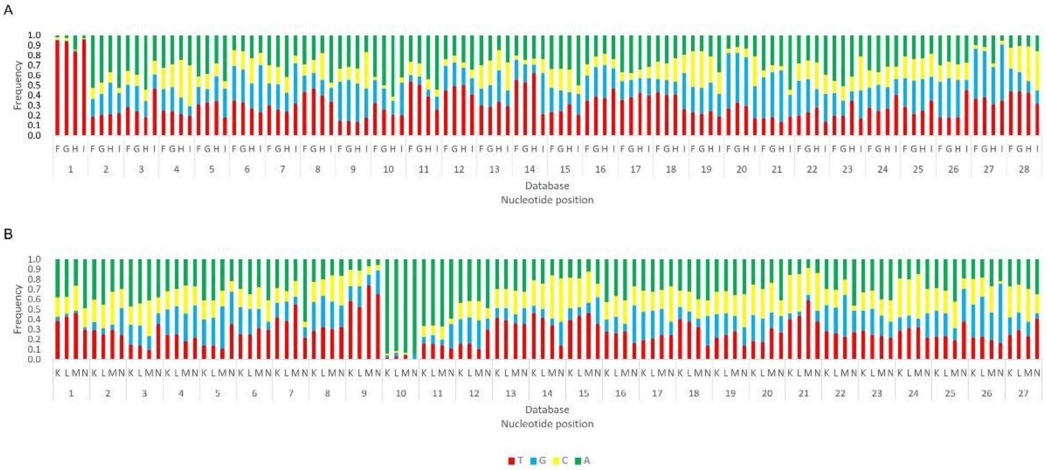

Figure S8: Relative nucleotide frequency of BmN4 sRNAs mapped to Aquila: antisense 28nt-long sRNAs bound to Siwi (A) and sense 27nt-long sRNAs bound to Ago3 (B), databases F-N (Table S2). The Y-axis represents the relative nucleotide frequency. The X-axis represents the position in the read. T: thymine; G: guanine; C: cytosine; A: adenine. Since the sequenced reads correspond to cDNA, thymine corresponds to uracil in the original RNA molecule.

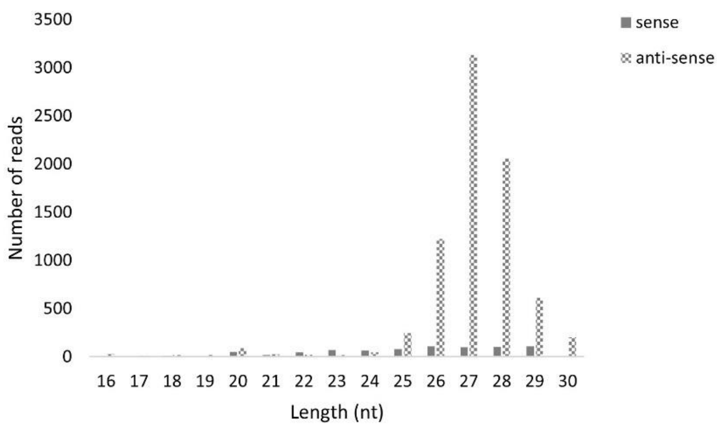

Figure S9: Length distribution of Tn5B sRNAs in High Five cells. The Y-axis represents the number of reads mapped to the transposable element Tn5B. The X-axis represents the length of the reads in nucleotides. Solid bars: sense reads. Dotted bars: anti-sense reads.
